# Grafted human induced pluripotent stem cells improve the outcome of spinal cord injury: modulation of the lesion microenvironment

**DOI:** 10.1038/s41598-020-79846-2

**Published:** 2020-12-29

**Authors:** Tamás Bellák, Zoltán Fekécs, Dénes Török, Zsuzsanna Táncos, Csilla Nemes, Zsófia Tézsla, László Gál, Suchitra Polgári, Julianna Kobolák, András Dinnyés, Antal Nógrádi, Krisztián Pajer

**Affiliations:** 1 Department of Anatomy, Histology and Embryology, Faculty of Medicine, University of Szeged, Kossuth Lajos sgt. 40., 6724 Szeged, Hungary; 2 BioTalentum Ltd., Gödöllő, Hungary; 3 HCEMM-USZ StemCell Research Group, Szeged, Hungary; 4 Department of Dermatology and Allergology, Research Institute of Translational Biomedicine, University of Szeged, Szeged, Hungary; 5 Present Address: Department of Diagnostic Laboratory, State Health Centre, Military Hospital, Budapest, Hungary

**Keywords:** Neuroscience, Regeneration and repair in the nervous system, Spinal cord injury

## Abstract

Spinal cord injury results in irreversible tissue damage followed by a very limited recovery of function. In this study we investigated whether transplantation of undifferentiated human induced pluripotent stem cells (hiPSCs) into the injured rat spinal cord is able to induce morphological and functional improvement. hiPSCs were grafted intraspinally or intravenously one week after a thoracic (T11) spinal cord contusion injury performed in Fischer 344 rats. Grafted animals showed significantly better functional recovery than the control rats which received only contusion injury. Morphologically, the contusion cavity was significantly smaller, and the amount of spared tissue was significantly greater in grafted animals than in controls. Retrograde tracing studies showed a statistically significant increase in the number of FB-labeled neurons in different segments of the spinal cord, the brainstem and the sensorimotor cortex. The extent of functional improvement was inversely related to the amount of chondroitin-sulphate around the cavity and the astrocytic and microglial reactions in the injured segment. The grafts produced GDNF, IL-10 and MIP1-alpha for at least one week. These data suggest that grafted undifferentiated hiPSCs are able to induce morphological and functional recovery after spinal cord contusion injury.

## Introduction

Spinal cord injury (SCI) is a devastating neurological condition with very limited recovery of function, which diminishes the quality of life both the patient and his/her family^1–3^. Treatment opportunities are limited and often revolve around preventing further damage with interventions involving rehabilitation. The pathological changes following SCI involve a primary and a secondary phase. The primary phase is the development of the initial injury caused by the impact followed by a rapid and progressive secondary injury cascade. In this phase of SCI a cascade of processes, such as oedema, neuroinflammation and excitotoxicity results in glial and neural death^4^.

Cell transplantation is one of the most promising strategies to induce functional improvement in SCI^5,6^. Many cell-based therapies have utilized various types of stem cells. Numerous stem cells and their derivatives have been reported to modify the lesion environment and induce some regeneration of damaged neurons, remyelination of axons, trophic support or a combination thereof^7–10^. The use of human embryonic stem cell-based therapies often raised ethical issues. Moreover, they proved to be allogenic, leading to immune rejection or requiring lifetime immunosuppression. The establishment of human induced pluripotent stem cells (hiPSCs) opened new ways on the horizon of regenerative therapies. The iPSCs can be produced from somatic cells such as dermal fibroblasts, keratinocytes or blood cells by transient overexpression of defined transcription factors, such as Oct3/4, Sox2, Klf4, and c-Myc (known as OSKM factors)^11,12^. Human iPSCs display similar morphology, proliferation capacity, surface antigens expression, gene expression characteristics as embryonic stem cells^11–14^. Moreover, transplantation of iPSC derivatives reportedly does not generate significant host immune response^15,16^. Various iPSCs-derived progenitors have been grafted into the injured spinal cord and considerable information has been gained about their survival, proliferation, and unique differentiation profile following transplantation^16–21^. Nevertheless, more preclinical studies have yet to be performed to investigate the regenerative potential and safety of hiPSCs. In our study we used the SB5 hiPSC line that exhibits the characteristics of pluripotent stem cells, including the expression of embryonic stem cell markers and has the ability to differentiate in vitro into the three germ layers as we published before^22^. Based on the properties of the SB5 hiPSC line, we hypothesized that transplantation of these cells into a contused spinal cord may lead to considerable morphological regeneration/tissue sparing and restoration of function after injury.

The aim of the present study was to investigate whether transplantation of undifferentiated hiPSCs into the injured rat spinal cord may lead to secretion of a set of bioactive molecules—called “lesion induced secretome”—which may support the repair of damaged tissue by modulating the local immune response, enhancing tissue sparing and altering the lesion microenvironment to support axonal regeneration/plasticity.

## Results

### In vitro characterization of SB5 hiPSC line

hiPSCs were characterized in vitro for pluripotency markers and their differentiation capacity. All colonies expressed the pluripotency markers NANOG, OCT3/4, SOX2 and SSEA-4 (Supplementary Fig. S1A). The hiPSCs were able to differentiate spontaneously into each embryonic germ layer with high efficiency based on the expression of GATA4, PDX1 (endoderm), Brachyury, Tropomyosin (TPM2) (mesoderm), Musashi-1 (MSI1), NESTIN, TUBB3 and MAP2 (ectoderm) as confirmed with immunohistochemical analysis (Supplementary Fig. S1B).

Cell viability assessment was performed before transplantation. Five minutes after harvesting the hiPSCs 100% cell viability was detected. Twenty-five minutes later 99% of the cells proved to be viable (Supplementary Fig. S2).

### Hind limb locomotor pattern has been improved following intraspinal transplantation of hiPSCs

**Figure 1:**
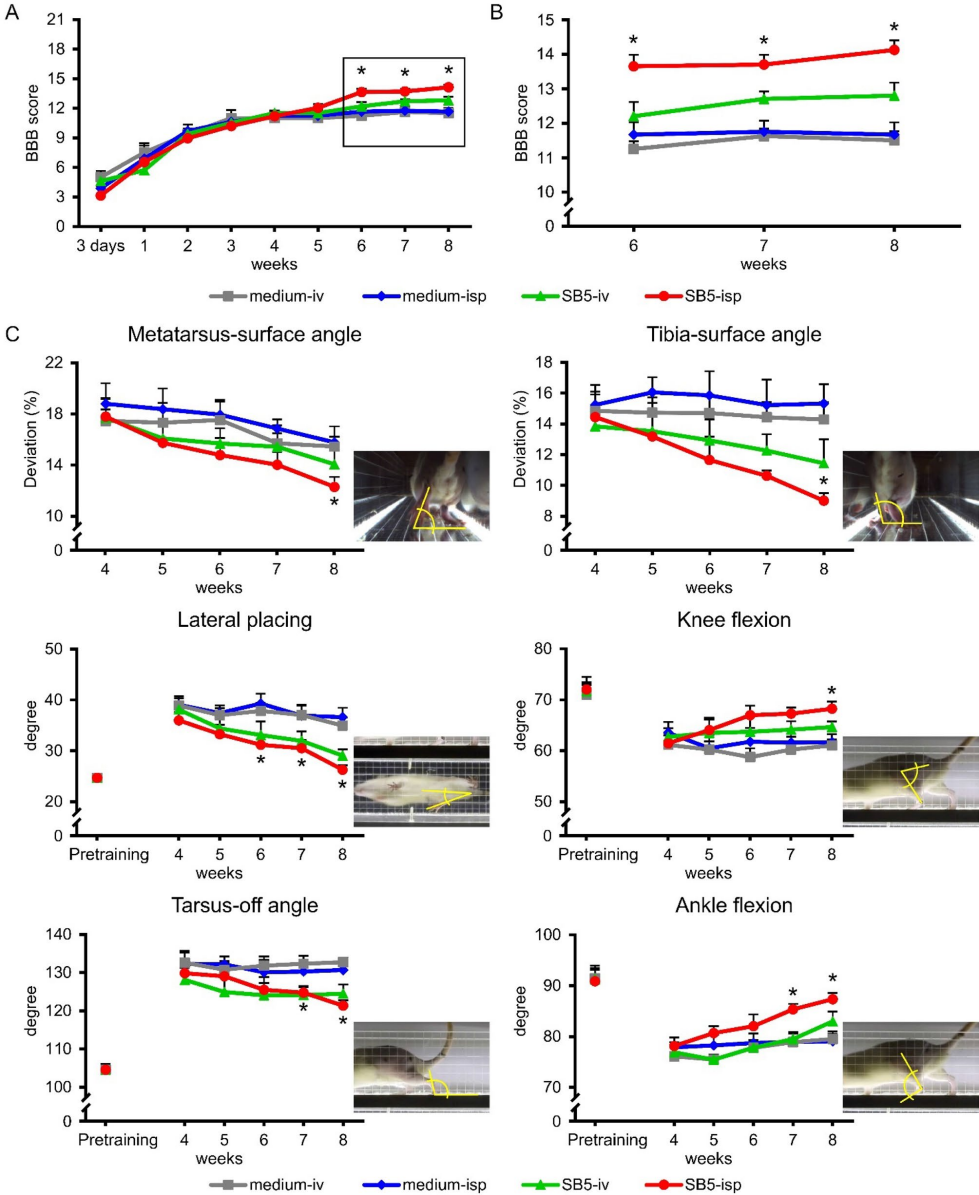
Intraspinal hiPSC transplant improves locomotor function. (**A**) Open field locomotor test (BBB) shows significant improvement of intraspinally grafted animals (*SB5-isp*) compared with their controls, although the *SB5-iv* animals showed no significant recovery of their locomotor pattern from week 6 onwards. (**B**) Enlarged view of the graphs in A from week 6 onwards. Asterisks represent significant difference between intraspinally grafted (*SB5-isp*) group and its control group at various time points. (**C**) Kinematic analysis of the animals in the various groups after injury. Note the significantly improved parameters of the grafted animals (*SB5-isp*) compared with their controls. Data are expressed as mean ± SEM (n = 8 in each group) *significant difference between the intraspinally grafted animals (*SB5-isp*) and its control (*medium-isp*). *p* values: BBB-test (*p* = 0.012 at week 6, *p* = 0.022 at week 7, *p* = 0.016 at week 8), metatarsus-surface angle (*p* = 0.048 at week 8), tibia-surface angle (*p* = 0.046 at week 8), lateral placing (*p* = 0.047 at week 6, *p* = 0.049 at week 7, *p* = 0.002 at week 8), knee flexion (*p* = 0.048 at week 8), tarsus-off angle (*p* = 0.005 at week 8), ankle flexion (*p* = 0.036 at week 7, *p* = 0.003 at week 8).

Hind limb locomotor recovery was assessed using the BBB open field test and kinematic analysis measures (Fig. 1). During the first week, injured rats showed no weight supported stepping. At week 1, injured rats were randomized into four experimental groups to ensure equivalent deficits across the groups. The animals showed frequent to consistent weight supported plantar steps and occasional consistent weight supported plantar steps at week 5 in all groups. At weeks 6 and 7 the stem cell-treated animals (*SB5-iv*, *SB5-isp* groups) showed a slight increase in BBB score compared to their controls. Using the two-way repeated measures of ANOVA from 1 to 9 weeks after injury, we found a statistically significant interaction only between the *SB5-isp* and their controls at weeks 6–8 after injury (*p* < 0.05, n = 8 in each group). These rats showed consistent weight supported plantar steps and consistent fore- and hind limb coordination (Fig. 1A,B).

The kinematic analysis of transplanted and control rats was evaluated for metatarsus surface angle, tibia-surface angle, lateral placing, knee flexion, tarsus off angle and ankle flexion. During pre-injury training, all rats accurately accomplished the test. After SCI, all the four groups (*medium-iv*, *medium-isp*, *SB5-iv* and *SB5-isp* animals; n = 8 in each group) demonstrated deficits in hindlimb placements. Consistent with the BBB results, all groups showed a non-significantly improvement up to week 5. From week 6 onwards the rats that received hiPSCs treatment intraspinally (*SB5-isp*) progressively improved until week 8 (Fig. 1C). The kinematic analysis assessments also revealed that the *SB5-isp* animals displayed a consistent improvement in the metatarsus surface angle, tibia-surface angle, lateral placing knee flexion, tarsus off angle and ankle flexion in hindlimb placement in contrast to control animals that displayed slight recovery after SCI. Only the intraspinally grafted animals (*SB5-isp*) were able to approach the intact pre-training values and showed statistically significant improvement compared with their controls (Fig. 1C).

### Intraspinally grafted hiPSCs enhance tissue sparing

**Figure 2:**
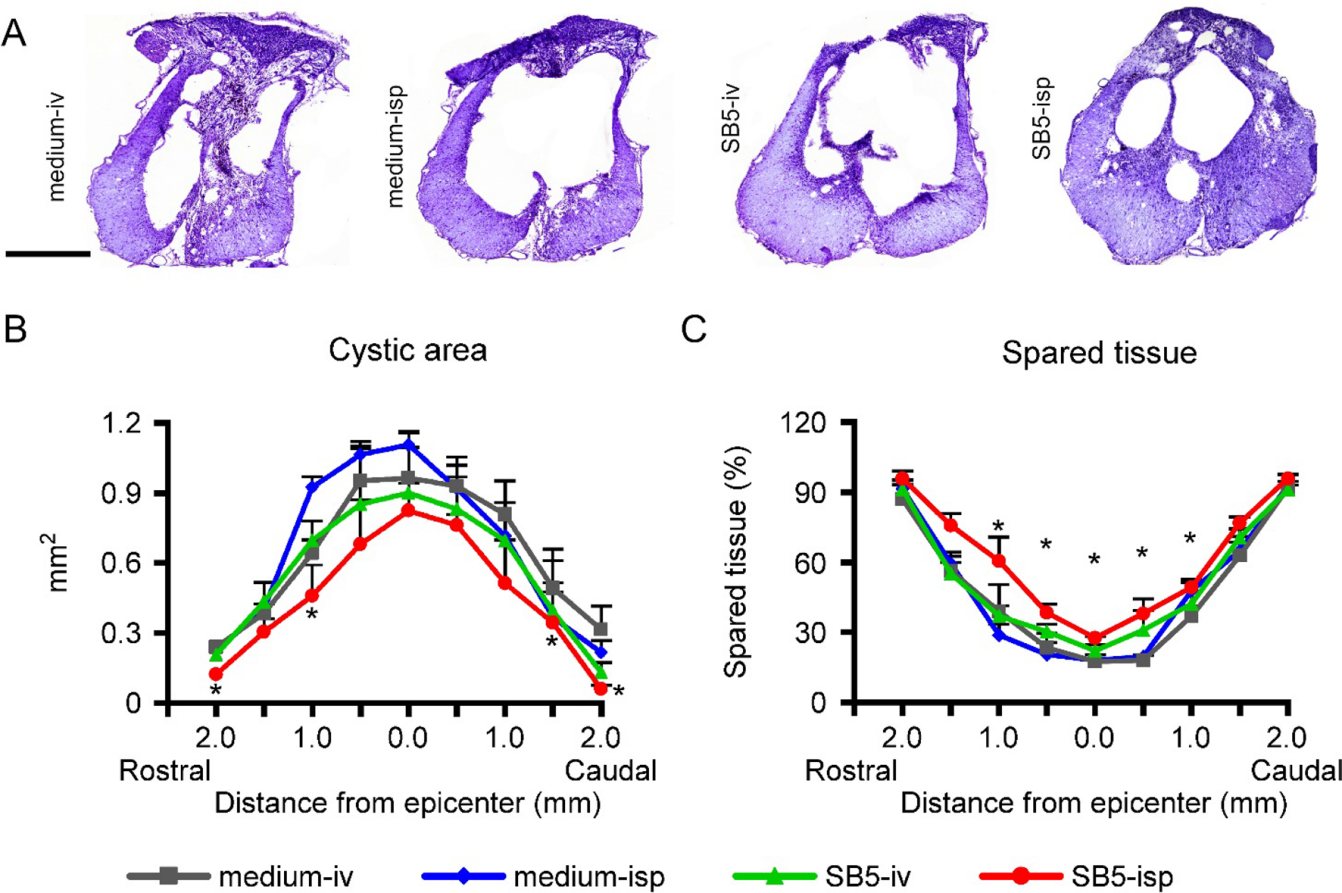
Intraspinally grafted hiPSCs promote tissue sparing. (**A**) Representative images taken at 100 μm rostral to the SCI lesion epicentre. (**B**) Quantification of cystic area shows that intraspinal hiPSC treatment resulted in considerably reduced size of injury following SCI. (**C**) Improved tissue sparing is seen rostro-caudally to the injury epicenter in the intraspinally grafted group (*SB5-isp*). Significantly more tissue sparing was detected in the intraspinally treated group (*SB5-isp* group) compared with its control (*medium-isp*). Data are expressed as mean ± SEM. (n = 8 in each group) *significant difference between the intraspinally grafted animals (*SB5-isp*) and its control. Scale bar: 500 µm.

Histomorphometric analysis was performed to quantify spinal cord tissue changes 9 weeks after SCI (n = 4 in each group) (Fig. 2). Within the injured segment a large, centrally located cystic cavity was formed containing cellular debris or trabecula. The greatest amount of intact-looking white matter localized to the ventral and ventrolateral parts of the spinal cord in the experimental groups (Fig. 2A). Reduced cystic tissue was observed in the epicenter and 0.5 mm rostrally and caudally to the injury epicenter in grafted animals. Significantly decreased lesion area was identified at 1 mm and 2 mm rostrally and at 1.5 mm and 2 mm caudally to injury in the spinal cord of animals in the *SB5-isp* group (*SB5-isp* vs. *medium-isp* group, *p* < 0.05; Fig. 2B). Quantification of spared tissue in injured spinal cords based on cresyl-violet staining indicated that a significantly greater amount of tissue was preserved in the animals of the intraspinally grafted group compared with its controls (*SB5-isp* vs. *medium-isp* group, *p* < 0.05; Fig. 2C).

### hiPSCs treatment leads to improved connections as revealed by retrograde tracing

**Figure 3:**
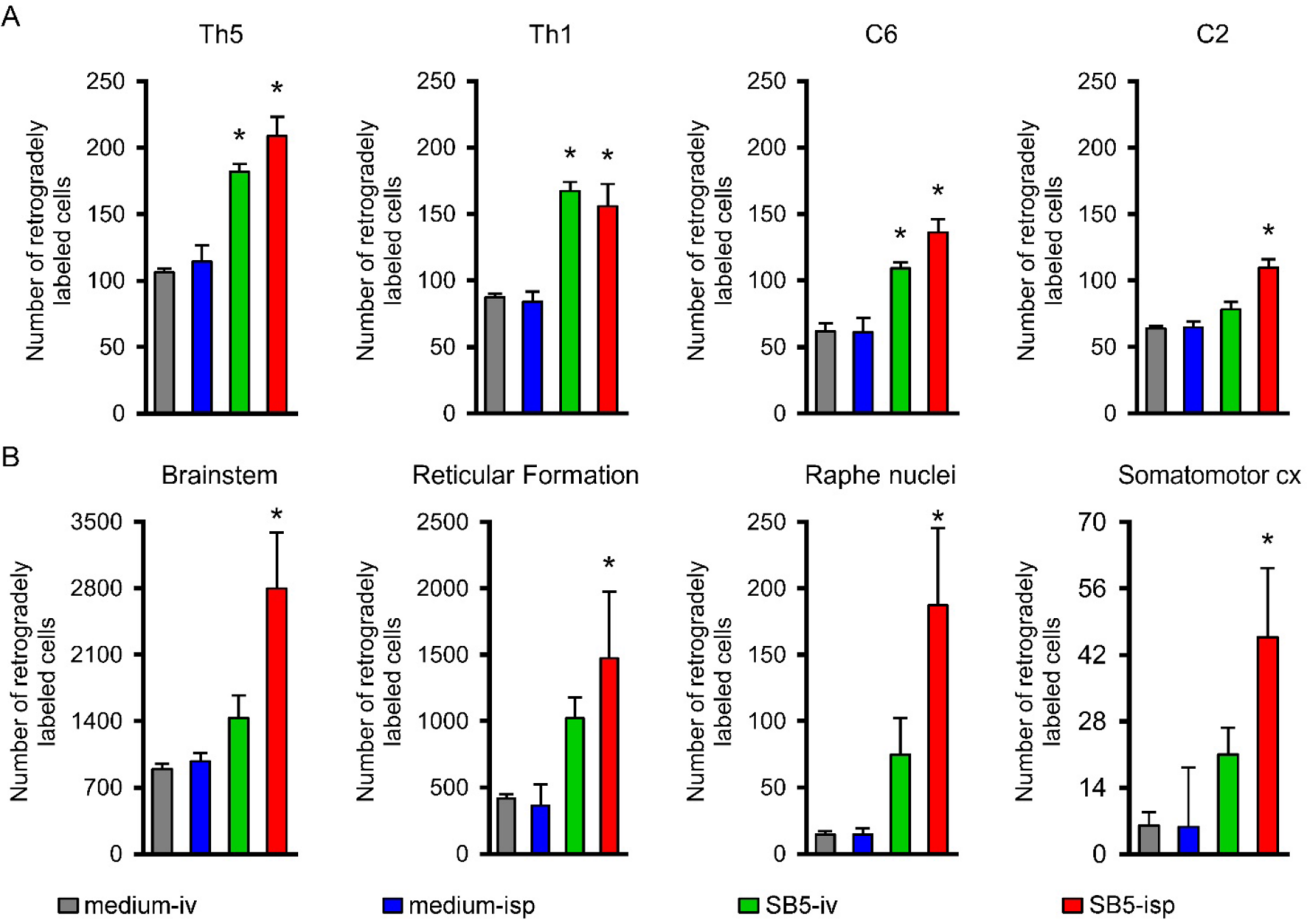
Morphometric analysis of the connections between the segments caudal to the lesion and various cranial parts of the CNS. (**A**) Numbers of neurons retrogradely labeled from the right L3 hemisegment are shown. Note the significantly higher numbers of labeled cells in the spinal cord of the hiPSC treated animals (*SB5-iv*, *SB5-isp*) compared with their controls (*medium-iv*, *medium-isp*). The greatest number of retrogradely labeled cells was always found in the intraspinally treated animals. (**B**) Significantly greater numbers of traced neurons are found in the brainstem, reticular formation, the raphe nuclei and the somatomotor cortex of intraspinally transplanted animals as compared with their controls. Note the limited regenerative capacity in the case of intravenous delivery of stem cells. Data are expressed as mean ± SEM. (n = 4 in each group) *significant difference between the intraspinally or intravenously grafted animals with the control ones in A. *significant difference between the intraspinally grafted animals and its control in B.

Next, we evaluated whether axonal regeneration/sparing was promoted by the grafted hiPSCs. Labeling of propriospinal and supraspinal neurons in the spinal cord and brain was evaluated by placing the retrograde tracer (Fast Blue) caudally to the injury into the right L3 segment, the numbers of retrogradely labeled neuronal somata in the spinal cord, brainstem and brain were determined (n = 4 in each group). These data refer to the number of neurons above the injury site which axons take up the tracer in the caudal spinal cord segment. Significantly higher numbers of FB-labeled propriospinal neurons were found in the Th5, Th1, C6 and C2 spinal segments in animals treated with hiPSCs (both *SB5-iv* and *SB5-isp*) than in their controls (Fig. 3A, Supplementary Fig. S3A–E). It should be noted that the number of retrogradely traced neurons decreased with the distance from the labeled segment.

We also identified retrogradely labeled neurons in the brainstem (particularly in the reticular formation, raphe nuclei) and in the somatomotor cortex. Following intraspinal implantation of hiPSCs significantly higher number of retrogradely labeled neuronal somata were found in the different brain regions. In the case of systemic (iv) delivery of hiPSCs (*SB5-iv*), non-significantly higher number of FB + neurons were found in these supraspinal locations compared with their controls (Fig. 3B).

### hiPSC treatment promotes preservation/sprouting of serotonergic fibers caudally to the injury

Since hiPSC treatment promoted tissue/axonal sparing, we further examined the descending serotonergic pathway, which is a multiple descending tract from the brainstem and is known to regulate the spinal locomotion^23,24^. In the control group (*medium-isp*), 5-HT-positive fibers could be observed in close vicinity to the rostral end of cavity (Supplementary Fig. S4A–C). Numerous aborted endings could be detected around the lesion, suggesting the abortive regeneration of 5-HT-positive fibers (Supplementary Fig. S4C′). In the *SB5-isp* group we observed sprouting of the 5-HT-positive fibers rostrally to the cavity in a small boundary while serotonergic innervation of certain neurons was well preserved (Supplementary Fig. S4B-D′). Caudal to the lesion, we also found 5-HT-positive fibers in both groups (*medium-isp, SB5-isp*), although to a lesser extent than rostrally to the injury. In control animals, the presence of serotonergic fibers was weaker caudally to the lesion compared to treated rats (Supplementary Fig. S4E–H′). In grafted animals, considerable higher density of 5-HT-positive fibers could be found caudally to injury (Supplementary Fig. S1F–J′). Close to caudal end of the lesion (500 µm away from the caudal end), animals in the SB5-isp group showed 3.31 ± 1.42 fold increase in 5-HT expression compared to injured value (Supplementary Fig. S4K). The level of 5-HT expression in the spinal cords of the SB5-isp group also remained significantly higher than in the control group 1000 and 1500 µm caudally to the lesion (Supplementary Fig. S4K). The striking difference in the density of 5-HT-positive fibers between the groups may be due to preservation of serotonergic axons and it was likely supported by hiPSC treatment.

### hiPSC treatment influences the astroglia and microglia/macrophage reaction

**Figure 4:**
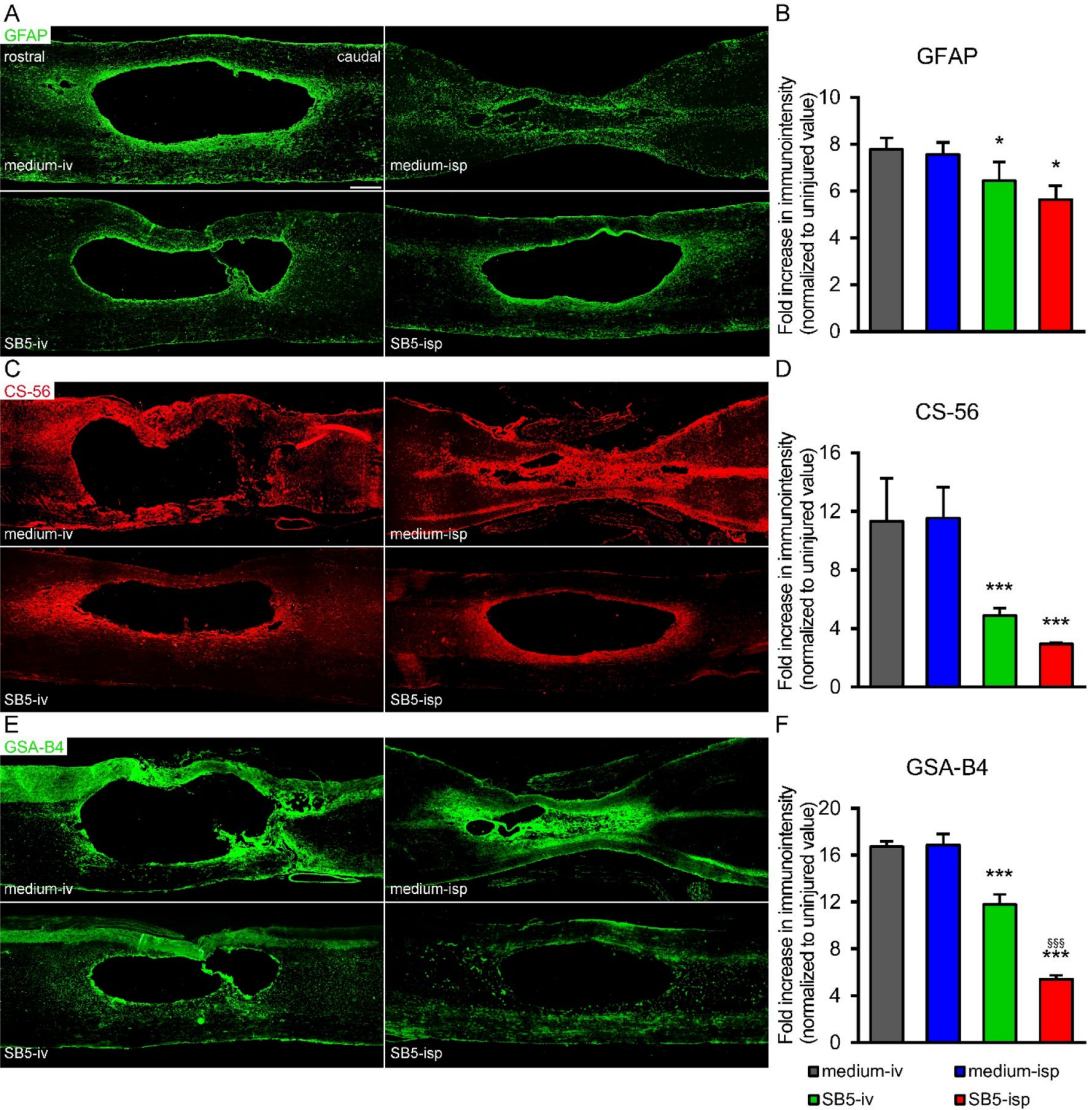
Modulation of the lesion environment. (**A**,**C**,**E**) Low magnification images of parasagittal spinal cord sections show the GFAP, CS-56 and GSA-B4 reactivity 9 weeks after the injury around the lesion area in the various experimental groups. (**B**,**D**,**F**) Quantification of GFAP, CS-56 and GSA-B4 reactivities in the sagittal sections of the spinal cord revealed a decreased level of all examined markers in hiPSC-treated groups (*SB5-iv*, *SB5-isp*) compared with the control groups (*medium-iv*, *medium-isp*). Data are expressed as mean ± SEM (n = 4 in each group). *p < 0.05 *significant difference between the intraspinally (*SB5-isp*) or intravenously (*SB5-iv*) grafted animals and the control animals in B. ***p < 0.01 ***significant difference between the intraspinally or intravenously grafted animals with the control animals in D and F. ^§§§^*p* < 0.01 ^§§§^significant difference between the intraspinally grafted animals (*SB5-isp*) and the intravenously treated animals (*SB5-iv*) in D and F. Scale bar in A: 200 µm.

Next, we investigated whether the hiPSC treatment has the potential to alter the microenvironment of the lesion rendering it permissive for regenerating axons. Therefore, we examined and quantified the densities of astrocytes and microglia/macrophages and the deposition of chondroitin sulphate proteoglycan (CSPG) 9 weeks after the injury around the lesion cavity (n = 4 in each group). Analysis of the immunostained sections showed differences in the amount of GFAP, CS-56 and GSA-B4 expression among the groups (Fig. 4A,C,E). Both intraspinal and intravenous application of hiPSCs induced significant reduction of astrocytosis indicated by significantly lower GFAP densities in these cords compared with controls (*SB5-isp*, *SB5-iv* vs. their controls, *p* < 0.05, Fig. 4B). While hiPSC treatment altered the GFAP immunointensity, marked reduction of CS-56 immunoreactivity could be observed compared to controls (*SB5-isp*, *SB5-iv* vs. their controls, *p* < 0.01, Fig. 4D). Similarly, microglia/macrophages in the lesion sites of grafted animals showed significantly reduced microgliosis (GSA-B4, lectin histochemistry) compared with the control groups (*SB5-isp*, *SB5-iv* vs. their controls, *p* < 0.01 Fig. 4F). However, grafting of hiPSCs induced significantly decreased microglia/macrophage densities at week 8 after grafting compared with the intravenous hiPSC treatment (*SB5-isp* vs. *SB5-iv*, *p* < 0.01).

### Intraspinally grafted iPSCs differentiate preferentially along a neuronal lineage

**Figure 5:**
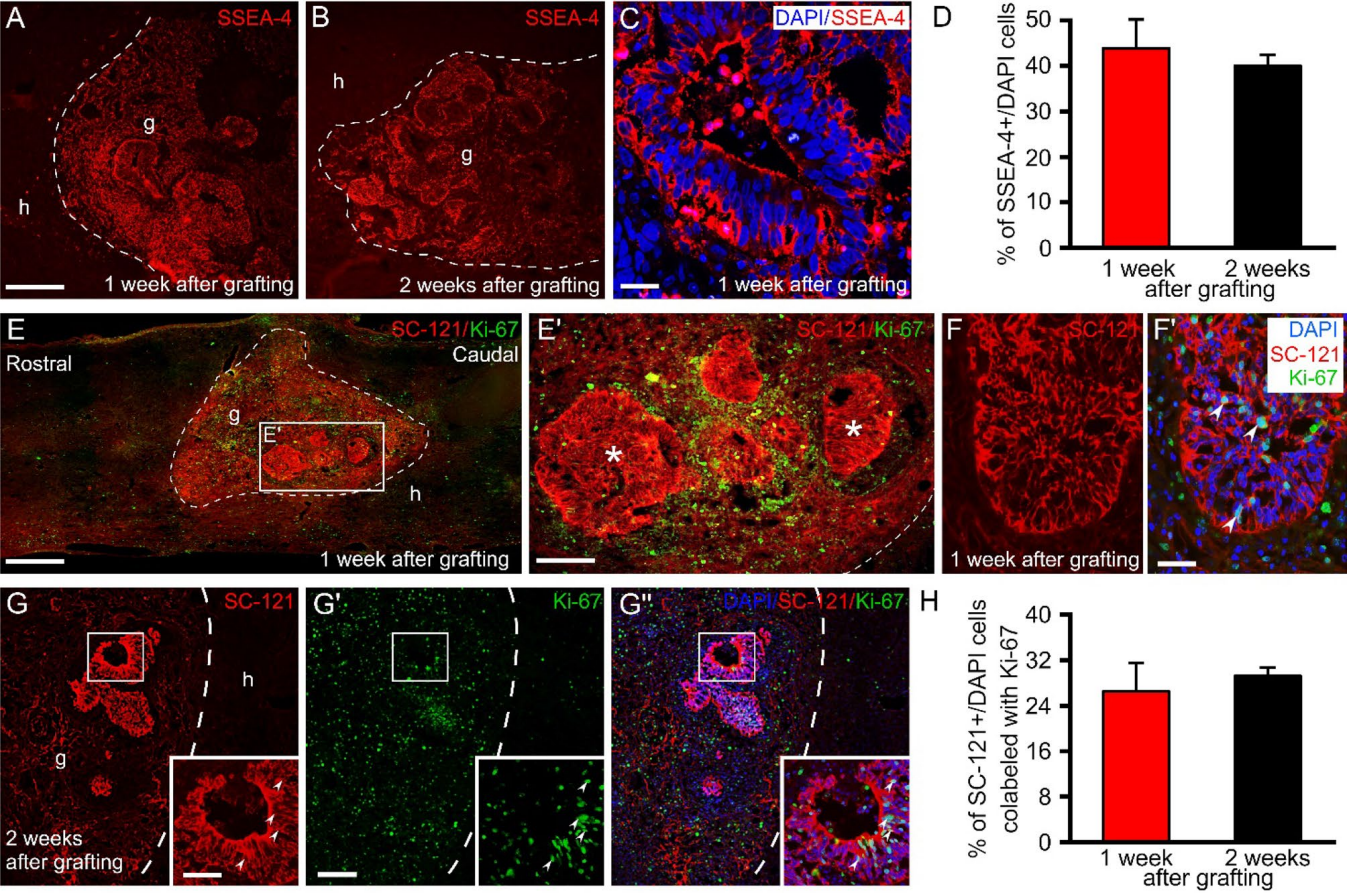
Transplanted hiPSCs proliferate within the injured spinal cord. Low and high-power images show the extent of hiPSC survival within the injured spinal cord 1 and 2 weeks after transplantation. (parasagittal sections **A**–**G**′′). (**A**–**C**) Expression of the embryonic stem cell marker SSEA-4 by the grafted hiPSCs 1 and 2 weeks after transplantation. (**D**) Cell quantification proved that the engrafted hiPSCs had a pluripotency fate identified by SSEA-4 expression (44.5% ± 5.1 and 40% ± 2.4; 1 week or 2 weeks after grafting, respectively). (**E**) The grafted hiPSCs displayed high Ki-67 positivity in the contusion cavity 1 and 2 weeks after grafting. (**E**′) Some engrafted hiPSCs form clusters (asterisks) while others remained dispersed. The enlarged figures in F and F′ show the grafted hiPSCs (SC-121 + , in red) with their DAPI-labeled nuclei, out of these many display Ki-67 positivity (green). (**G**–**G**′′) The transplanted cells (SC-121, red) showed high proliferative capacity (Ki-67, green) 2 weeks after transplantation. Arrowheads in the boxed area point to Ki-67 + nuclei associated with SC-121 + cells within the cluster. (H) Bar diagram shows the proliferative activity of grafted hiPSCs. Note that 26.5% ± 5.5 and 29.2% ± 1.5 (1 week or 2 weeks after grafting, respectively) of SCS-121 + hiPSCs were positive for Ki-67. Data are expressed as mean ± SEM. (n = 4 in each time point) **h**, host; **g**, graft. Scale bars: (**A**) 250 µm, (**C**) 25 µm, (**E**) 500 µm (**E**′) 250 µm, (**F**′) 50 µm, (**G)** 25 µm, (**G**′) 250 µm.

**Figure 6:**
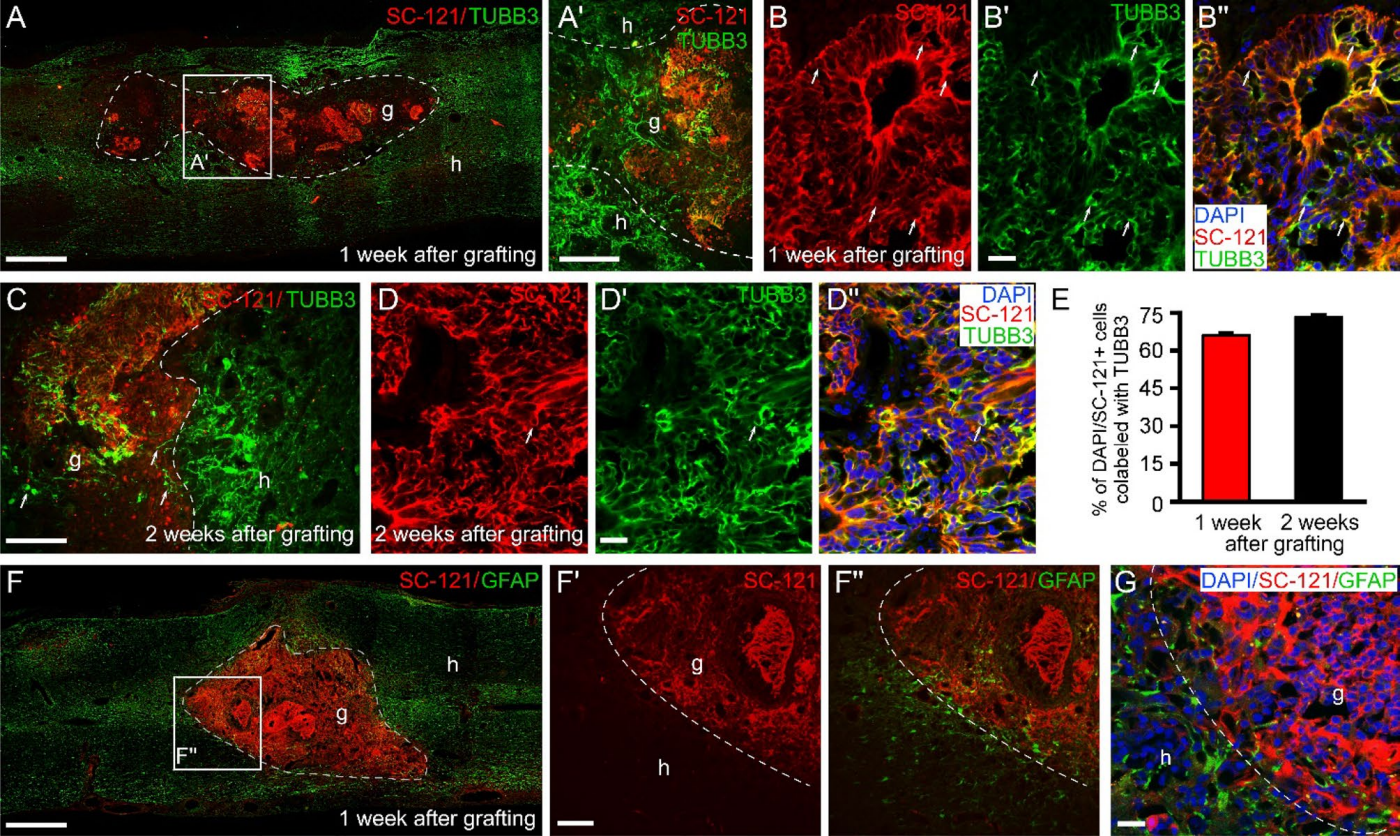
Neuronal differentiation of the intraspinally grafted hiPSCs in the injured rat spinal cord 1 and 2 weeks after transplantation. (**A**,**A**′) Differentiation of intraspinally grafted hiPSCs in the contusion cavity 1 week after grafting leads to the appearance of stem cell-derived neuronal populations in the graft. Host neurites around the lesion site were localized within or very close to the graft. (**A**′) Boxed area taken from the graft region shows the mixture of neurites of both host and graft origin. (**B**–**B**′′) Confocal images demonstrate differentiation of hiPSCs (red) along a neuronal lineage identified by TUBB3 (green) expression. (**C**) Notable TUBB3 (green) immunoreactivity was observed within the graft 2 weeks after grafting. (**D**–**D**′′) Colocalization of hiPSCs (red) cells with TUBB3 protein (green) 2 weeks after transplantation. (**E**) Our quantitative analysis 1 and 2 weeks after transplantation showed that the grafted cells mainly expressed TUBB3 (66.1% ± 6.7 and 73.3% ± 5.5, one and two weeks after grafting, respectively; n = 4 in each time point). (**F**–**F**′′) The grafted hiPSCs did not show any GFAP immunoreactivity 1 week after grafting. GFAP-positive immunoreactivity can be seen among the GFAP negative transplanted cells close to the host—graft interface. (**G**) Higher magnification clearly shows presence GFAP-positive processes among the grafted hiPSCs. Dashed line indicates the graft-host border. In **A**–**B**′′ and **D**–**D**′′ arrows show SC-121-positive grafted cells (red) that were colocalized with TUBB3 (green) and TUBB3 + host-derived neurites in (**C**). Data are expressed as mean ± SEM. (n = 4 in each group) g, graft, h, host; Scale bars: (**A**): 500 µm, (**A**′): 250 µm, (**B**′): 25 µm, (**C**) 250 µm, (**D**′): 25 µm, (**F**) 500 µm, (**F**′) 100 µm; (**G**) 10 µm.

To examine the feasibility of hiPSCs transplantation as a therapeutic tool for SCI, we grafted the cells into an injury cavity 1 week after SCI. One, two, four and eight weeks after transplantation, immunohistochemical analyses were performed to examine the survival, migration, proliferation and differentiation patterns of the grafted hiPSCs in the injured spinal cords. hiPSCs were mapped for expression of human SC-121 (reacts with a human cytoplasmic protein) and SC-101 (reacts with any human cell nucleus) in adjacent sections. At these early survival times, the hiPSCs did not migrate yet away from the graft (Figs. 5 and 6).

The engrafted hiPSCs survived remarkably, expressed SSEA-4, proliferated within the injured spinal cord and formed clusters or showed disperse distribution patterns (Fig. 5A–G”). The intensity of SC-121 immunostaining was stronger within the clusters formed by grafted cells than in their vicinity (Fig. 5E–G). The proliferation capacity of the clusters was evaluated with Ki-67 immunostaining and appeared to be low (Fig. 5E′). In contrast, around the clusters more Ki-67 + cells were displayed at both survival time points (Fig. 5E′–F′). Quantitative analysis showed that 1 and 2 weeks after transplantation, 26–29% of the SC-121-positive and 29–33% of the SC-101-positive grafted cells were Ki-67 positive (n = 4 in each time point) (Fig. 5H, Supplementary Fig. S5).

Using cellular markers, we investigated the differentiation pattern of hiPSCs in the *SB5-isp* group at these early survival time points (Fig. 6). Our findings demonstrated that the majority of the transplanted hiPSCs differentiated along a neuronal lineage as demonstrated by TUBB3/SC-121 and TUBB3/SC-101 co-expression at both experimental time points (TUBB3/SC-121: ~ 66% at 1 week and ~ 73% at 2 weeks; TUBB3/SC-101: ~ 62% at 1 week and ~ 74% at 2 weeks; n = 4 in each time point; Fig. 6A–E, Supplementary Fig. S5). These findings suggest that grafted hiPSCs promote preferential differentiation toward the neuronal lineage. We did not observe any hiPSC-derived cells expressing GFAP 1 week after transplantation (Fig. 6F–G). We also examined whether grafted hiPSCs had the potential to myelinate the host axons after transplantation. Confocal microscopic imaging revealed no MOG immunoreactive grafted cells, indicating that grafted cells had limited if any potential to differentiate into myelinating oligodendrocytes (Supplementary Fig. S6A–B).

**Figure 7:**
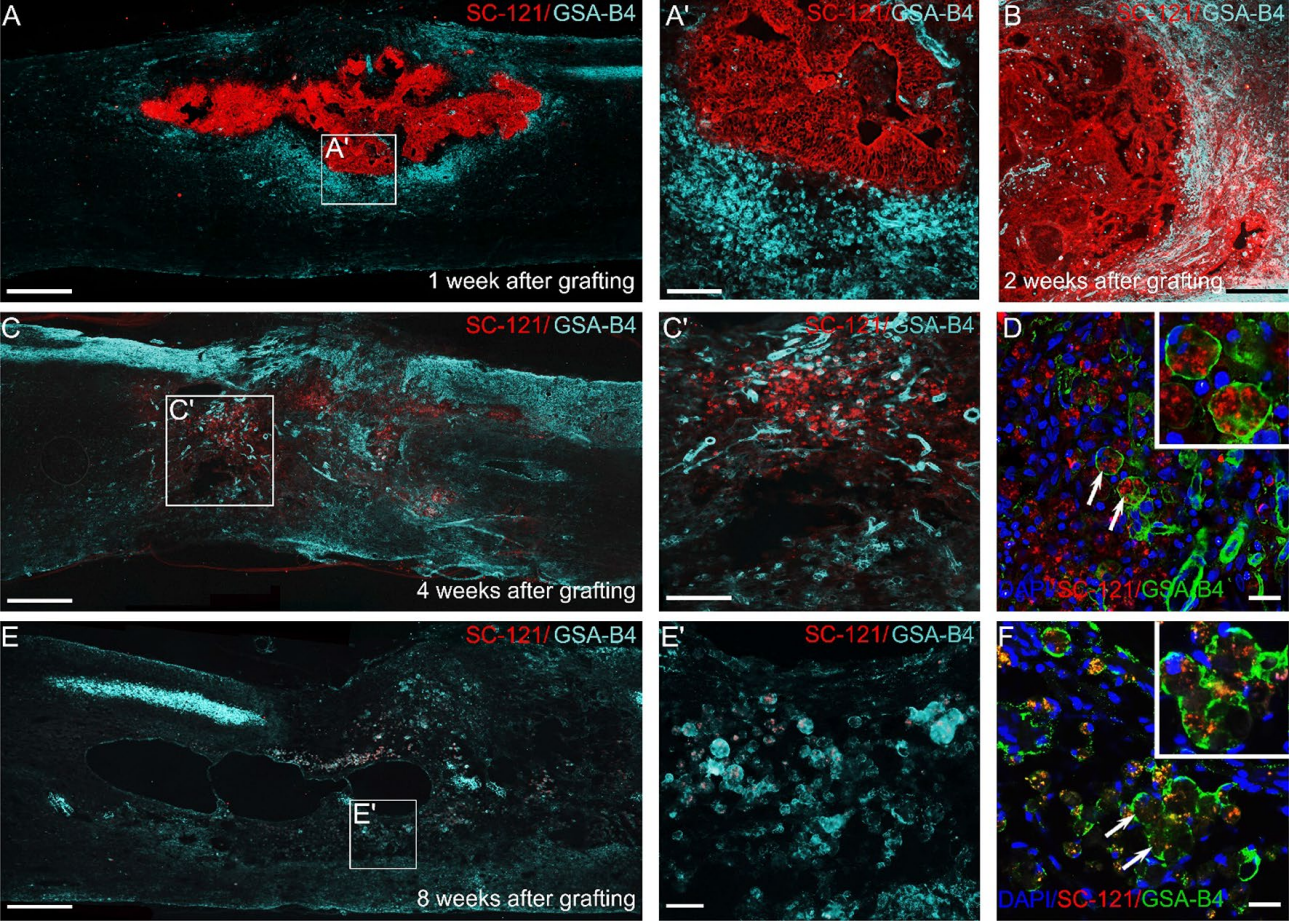
Microglia/macrophage reaction against grafted hiPSCs. (**A**,**A**′) Epifluorescence image of parasagittal longitudinal section of hiPSC treated spinal cords 1 week after intraspinal grafting shows the presence of GSA-B4-positive activated microglia/macrophages (blue) around the grafted area (red). (**B**) On week 2 after transplantation the activated microglia/macrophages showed higher density within the graft. (**C**,**C**′) On week 4 after grafting strong reactions of the activated microglia/macrophages could be detected in the grafted area. (**D**) Confocal immunostaining revealed intense engulfing of nonviable SC-121-positive profiles (red) by GSA-B4 positive macrophages/microglia. (**E**, **E**′) On week 8 after grafting decreased microglia/macrophage reactions were observed in the injured spinal cord. (**F**) Confocal image shows tiny SC-121-positive profiles in GSA-B4-positive cells. Scale bars: (**A**,**C**,**E**) 500 µm, (**A**′,**B**,**C**′) 100 µm, (**D**,**F)** 10 µm, (**E**′): 50 µm.

To further determine the fate of the grafted cell population, we used various markers (SC-121 and SC-101 for human cells, GFAP for astrocytes and TUBB3 for neurons) at 4 and 8 weeks after transplantation. At 4 weeks after hiPSC transplantation, sporadically observable SC-121-positive or SC-101-positive profiles were present in the grafted area (Supplementary Fig. S7). Interestingly, immunohistochemical analysis revealed intense TUBB3 and GFAP immunoreactivity associated with neurons and astrocytes surrounding the SC-121- or SC-101-positive profiles that had failed to express neuronal or glia marker. At 8 weeks after grafting, phagocytosed SC-121 + cellular fragments were readily observed in GSA-B4-positive macrophages (Fig. 7F).

### Clearance of intravenously delivered hiPSCs

To determine the fate of hiPSCs after intravenous administration (*SB5-iv*), tissue samples from certain organs—spinal cord, liver, lungs and spleen—were obtained 7 days after the intravenous transplantation. Grafted hiPSCs were mapped by SC-121 and DAPI co-staining under an epifluorescent microscope which allowed the determination of the presence or absence of the hiPSCs in the organs. In the examined time point no SC-121-positive cells were found in the various organs (Supplementary Fig. S8). This finding indicates the rapid clearance of the intravenously administered hiPSCs.

### Microglia/macrophage react to the grafted hiPSCs

In order to determine whether host microglia/macrophages invaded the territory of the grafted hiPSCs, a co-labeling with SC-121 immunostaining and GSA-B4 lectin histochemistry was performed in the instraspinally grafted group (*SB5-isp*). Seven days after grafting, increased activation of microglia/macrophages could be detected around the graft in the host cord (Fig. 7A,A′). Few GSA-B4-positive macrophages appeared at the graft-host interface suggesting that differentiation of the grafted cells exerts an attraction of macrophages to the cell differentiation zone (Fig. 7A,A′). Increased densities of the activated microglia/macrophages (GSA-B4-positive cells) could be observed in the graft 14 days after transplantation (Fig. 7B). Microglia/macrophage cells appeared around the graft-derived cells suggesting that the presence of antigens on the surface of grafted cells results in cell recognition and elimination by macrophages. On week 4 after grafting, the microglial/macrophage activity was significantly increased in the grafted area and a marked reduction of grafted cells were observed (Fig. 7C,C′). The majority of engrafted hiPSCs appeared as of nonviable SC-121-positive profiles and most of them appeared to have been incorporated by GSA-B4-positive cells (Fig. 7D). GSA-B4-positive cells could be observed in small groups around the cavity at 8 weeks after grafting (Fig. 7E,E′). The nonviable SC-121-positive cellular fragments could only be detected in GSA-B4-positive cells, suggesting that microglia/macrophages might be actively phagocytosing the grafted hiPSCs.

### Protein expression pattern of neurotrophic factors and cytokines in the grafted cells 1 week after transplantation

To determine the “secretome” of SB5 cells in vitro, first we analyzed the expression of 10 factors (IL-1-alpha, IL-6, IL-10, BDNF, GDNF, TNF-alpha, MIP-1-alpha, NT-4/5, VEGF, PDGFA), a selection based on our earlier results^25–28^. Strong immunoreactivity of GDNF, TNF-alpha and VEGF was found in vitro in undifferentiated SB5 cells (Supplementary Fig. S9).

**Figure 8:**
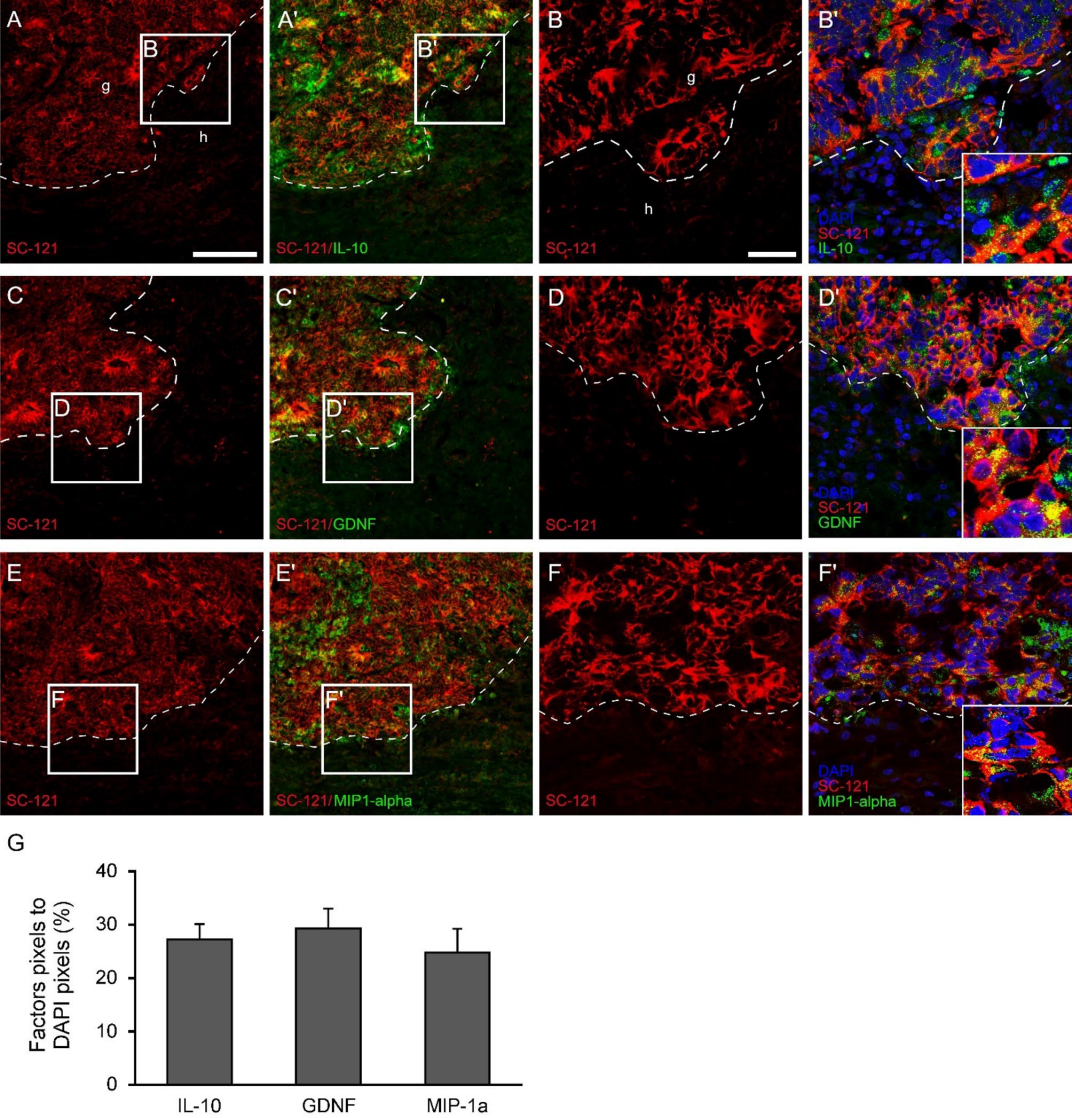
Expression of the various factors produced by the intraspinally grafted hiPSCs 1 week after grafting. Longitudinal sections of grafted spinal cord show the expression of IL-10, GDNF and MIP-1-alpha 1 week after transplantation within the graft. (**A**–**B**′,**C**–**D**′) The grafted hiPSCs expressed IL-10 and GDNF immunohistochemically detectable only in the graft. (**E**–**F**′) Relatively weak MIP-1-alpha expression could be observed within the graft. Higher magnification clearly shows the presence of factors in the cytoplasm in the boxed area of (**B**′,**D**′,**F**′). (**G**) Quantitative analysis of the immunostainings in fluorescence images. The numbers of IL-10, BDNF and MIP1-alpha immunoreactive pixels were normalized with DAPI positive pixels. Data were expressed as percentage of marker/DAPI ratio ± SEM. Scale bar: (**A**) 200 µm, (**B**) 100 µm.

Three out of the ten factors were found to be expressed in the graft (IL-10, GDNF and MIP-1-alpha) (Fig. 8). None of these factors was found to be expressed in the host spinal cords (Fig. 8A,A’C,C’E,E′). The expression of the factors was characterized by granular appearance. IL-10 showed similar distribution pattern to that of GDNF while weak MIP-1-alpha expression was confined to the grafted cells (Fig. 8B’D’E’G). Interestingly, controls or *SB5-iv* group showed no expression of the various factors within injured cords 2 weeks after the injury (*data not shown*).

### Increased TUBB3 and NF-200 expression around the graft

**Figure 9:**
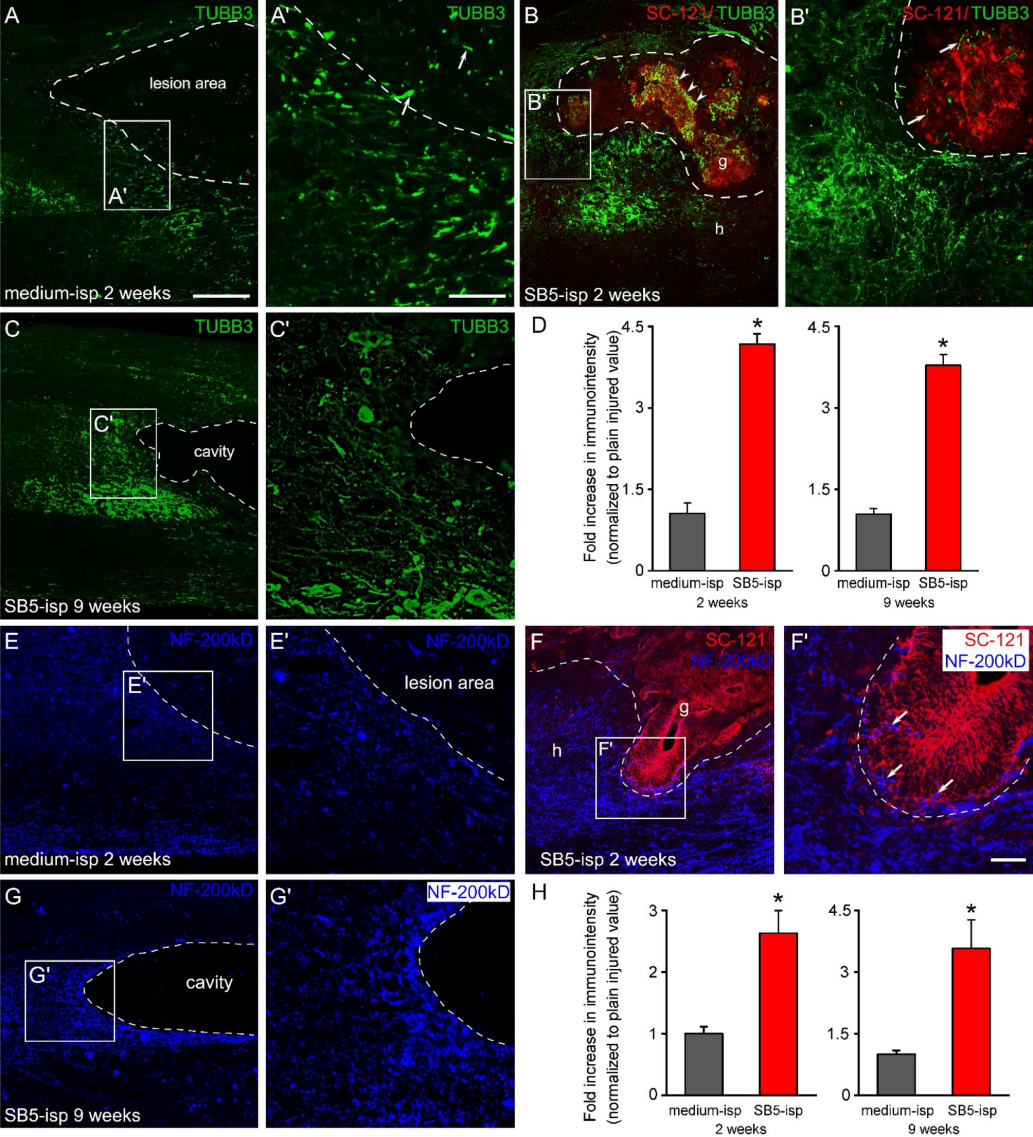
Increased TUBB3 immunoreactivity around the grafted area. (**A**–**B**) Representative images of TUBB3-positive host neurites in the control and grafted groups (*medium-isp and SB5-isp*) at the rostral part of the affected segment 2 weeks after the injury. (**A**′) TUBB3-positive host neurites tended to approach the lesion area in the control animal (*medium-isp*). (**B**′) TUBB3-positive host neurites display robust plasticity around and within the grafted area 1 week after hiPSC transplantation (*SB5-isp*). (**C**) A lesser extent of plasticity of TUBB3-positive host neurites was observed at 9 weeks after the injury in the grafted group (*SB5-isp*). (**D**) Quantification of TUBB3 immunointensity in the paramedian sagittal sections of the spinal cord revealed an increased level of immunoreactivity in hiPSC-treated group 2 and 9 weeks after the injury compared with the control group. (**E**, **E**′) Confocal images show NF-200kD-positive axons in the control group (*medium-isp*) 2 weeks after the injury. (**F**,**F**′) Numerous NF-200kD-positive host axons reached outside boundary of the graft. Quantification of NF-200 immunoreactivity demonstrated a significant increase in NF-200kD immunodensity in the grafted group 2 and 9 weeks after injury. Data are expressed as mean ± SEM. (n = 4 in both of the groups) *significant difference between the intraspinally (*SB5-isp*) with the control (*medium-isp*) animals in D and H. Dashed line indicates the lesion area/cavity area and spared tissue in (**A**,**A**′,**C**,**C**′,**E**,**E**′,**G**,**G**′ or the graft-host border in (**B**,**B**′,**F**,**F**′). Arrows show TUBB3-positive host neurites (in **A**′ and **B**′) and NF-200kD-positive host axons (in **F**′). Arrowheads point to TUBB3-positive grafted cells in B. g, graft; h, host; Scale bars: A: 200 µm, (A′) 100 µm, (F′) 30 µm.

Previous reports demonstrated an injury-induced plasticity within the injured spinal cord^29^. To test whether intraspinal hiPSC treatment exerts an effect on the density of TUBB3 immunoreactive fibers following SCI, we further examined the TUBB3 expression in the host tissue and the grafted area. The control group (*medium-isp*) showed moderate TUBB3 immunoreactivity around the lesion site 2 weeks after injury (Fig. 9A,A′). On the other hand, strong TUBB3 immunohistochemical reactivity was observed in the close vicinity of the grafted area in contrast to control animals suggesting that the increased plasticity of the host TUBB3-positive neurites was principally governed by grafted hiPSCs (Fig. 6A’C and Fig. 9A,B′). Numerous host neurites were also seen in close proximity of SC-121-positive cells (Fig. 9B,B′). A less dense TUBB3-positive fiber staining was apparent in grafted group (*SB5-isp*) around the cavity 9 weeks after the injury (Fig. 9C). Next, we measured the TUBB3 immunoreactivity in both groups (*medium-isp, SB5-isp*). Comparison of immunoreactivity of TUBB3 across the control (*medium-isp, 2 and 9 weeks after the injury*) and grafted (*SB5-isp, 2 and 9 weeks after injury*) experimental groups revealed a significant increase in TUBB3 expression (4.1 ± 0.19-fold increase in TUBB3 immunoreactivity in *medium isp 2 weeks* and 3.8 ± 0.19-fold increase in TUBB3 immunoreactivity in *medium isp 9 weeks*) in the host tissue of the grafted group (Fig. 9D).

We also evaluated the presence of NF-200kD-positive neurites in the host tissue around the lesion area. In the control group (*medium-isp*), NF-200kD-positive fibers appeared in the vicinity of lesion and only few axons could be observed within the lesion area 2 weeks after the injury (Fig. 9E,E′). In contrast, increased NF-200kD-positive immunoreaction were seen in grafted group (*SB5-isp*) 2 weeks after the injury. NF-200kD-positive fibers were also found at graft host interface, but these were not colocalized with the grafted cells, suggesting SC-121 + cells were not able to express NF-200kD (Fig. 9F,F′). At 9 weeks after the injury, grafted spinal cords (*SB5-isp*) showed slightly reduced NF-200kD expression (Fig. 9G,G′).

We next quantified the NF-200kD immunointensity around the lesion/grafted area. At 2 weeks after the injury, NF-200kD intensity was significantly increased in spinal cords of *SB5-isp* group (2.6 ± 0.36 fold increase) compared to *medium-isp* group (1.02 ± 0.11 fold increase; Fig. 9H). A considerably high level of NF-200kD immunointensity remained in grafted animals (*SB5-isp*) at 9 weeks after the injury (3.57 ± 0.70 fold increase). These findings suggest that factors produced by grafted hiPSCs increased the plasticity of TUBB3-positive host neurites and NF-200kD-positive host axons, respectively.

## Discussion

Our study provides strong evidence that intraspinal transplantation of undifferentiated hiPSCs is an effective strategy to induce tissue sparing after SCI. While a large body of evidence supports the therapeutic potential of iPSC-derived cell transplantation for subacute SCI^18,20,30,31^, grafting of undifferentiated hiPSCs into a lesioned spinal cord was not yet investigated. Our strategy, which uses undifferentiated hiPSCs transplants to decrease the inhibitory properties of the glial scar and microglia/macrophage reaction, successfully promoted the functional repair and plasticity of the spinal cord in a subacute SCI. The significant amount of spared tissue made it possible to recover the temporarily disrupted connections between the intact regions above and below the injury site. The considerable tissue sparing yielded a higher number of retrogradely labeled neurons in the spinal cord and the various brain regions of intraspinally grafted animals compared to the controls. Our locomotor assessments also showed a beneficial effect of intraspinal iPSC treatment on functional recovery.

The intraspinal grafting also preserved and promoted axonal sprouting including serotonergic fibers. The descending serotonergic tracts modulate directly the locomotor function^32^. Disruption of the serotonergic pathway following SCI prevents the activation of locomotor central pattern generator and results in a subsequent depletion in 5-HT^33^. We found that intraspinal hiPSC treatment rescued the serotonergic innervation of neurons caudal to the injury. This is in agreement with previous works that have shown that enhancing the plasticity of serotonergic fibers leads to improved recovery of locomotion^34,35^.

Previous studies have shown that intravenous cell treatment is a promising strategy to induce both morphological and functional recovery following subacute SCI^27,36–38^. Intravenous infusion of stem cells for SCI had a protective effect on blood vessels, reduced the area of spinal cord cavitation and promoted the restoration of motor function^27,37,39–41^. In this study, the intravenous application of hiPSCs was also able to reduce the microglia/macrophage reactions and deposition of chondroitin-sulphate, but no significant tissue sparing was observed in the affected segment. Interestingly, intravenous hiPSC application uniquely increased the number of retrogradely labeled neurons in the spinal cord. These results suggest that intravenously applied SB5 cells may exert their primary effects outside the spinal cord perhaps by altering the immune-mediated secondary pathological events after spinal cord injury. Despite the higher number of retrogradely labeled neurons and decrease of glia reaction and deposits of CSPGs in the injured spinal cords, we could detect only moderate locomotor recovery in these animals.

Similarly to other studies, the intravenously administered cells did not settle in the organs due to the rapid cell clearance^27,37^. It could be argued that this controversy between the morphological and functional data may be partly due to the poor survival of human cells in immune-competent animals or a higher dose of hiPSCs is required to improve the tissue-sparing after SCI. Further investigation is needed to elucidate the exact mechanism and effects of systemic hiPSC treatment on the injured spinal cord.

In our study, human fibroblasts can be reprogrammed using the Sleeping Beauty (SB) transposon system, offering an efficient, non-viral based method for producing hiPSCs. The hiPSCs are capable of generating all three embryonic germ layers when they are cultured in media containing serum. These results confirm the pluripotency of these cells that had been extensively investigated previously^22^. Furthermore, in vitro results have provided evidence for the production of GDNF, TNF-alpha and VEGF by undifferentiated SB5 hiPSCs.

The injury environment and cell density of grafted cells influence cell fate and differentiation^42–44^. The endogenous environment of the injured spinal cord may contain inhibitory factors that make the environment unfavorable for generating new neurons or myelinating oligodendrocytes from grafted cells^45–48^. In our study, the grafted cells were located in the lesion area and did not migrate away from the graft. The transplanted cells formed clusters of living cells within the graft area and approximately 27% of the grafted cells were positive for Ki-67. The low proliferative rate in the cell clusters combined with vigorous proliferation in the periphery of the graft suggested that the lack of cell migration and cluster formation may have induced extensive differentiation. Further analysis with neuronal markers (TUBB3) showed that approximately 75% of grafted cells differentiated into a neuronal lineage.

On the other hand, and in contrast to other studies no glial differentiation was found among the grafted human iPSCs. Although we clearly observed the close association of transplanted cells with endogenous astroglial processes at the graft-host border, no GFAP-positive grafted cells were detected. Our results suggest that the transplanted hiPSC failed to differentiate into mature myelin-forming oligodendrocytes, too. These observations suggest that the microenvironment of the injured spinal cord restricts the differentiation capacities of engrafted iPSCs and the cell source is crucial for the fate of the transplanted cells^49^.

The iPSC-derived neural stem/progenitor cells are characterized by low expression level of immune-related proteins and immunosuppressive effects. In vivo experiments have shown that the survival of such transplanted cells is higher in the injured spinal cord than in the intact environment^14,50^. Several attempts have been made to remove potentially tumorigenic cells before^51^ or after transplantation^52^, or promote the differentiation process of transplanted hiPSCs-derived neural stem/progenitor cells^47^. The optimal time of transplantation and the cell differentiation stage reportedly leads to the inhibition of cellular overgrowth, which may cause compression or destruction of the host tissue resulting in further motor deficits^53^. Tumorigenesis following hiPSC‐derived neural stem/progenitor cells was reported in murine SCI models^54,55^, but without the use of immunosuppressants the transplanted cells died after numerous weeks of survival. Interruption of immunosuppressive treatment resulted in the complete rejection of iPSCs-derived cell masses. Infiltration of microglia/macrophage cells and lymphocytes was observed during the course of rejection of tumor-like cells, along with apoptosis of iPSC-derived cells^52^. Similarly to these studies, our xenografted hiPSCs survived up to 2 weeks in the injured cord. In this early phase, the grafted iPSCs underwent a relatively fast differentiation process, accompanied by an intense and increasing microglial/macrophage response, resulting in elimination of the grafted iPSCs by week 4 after grafting. It could be argued that this fast cell differentiation is associated with the early exposure of cell surface antigens, leading to the rapid elimination of the grafted cells by the host immune system. These results are in agreement with our previous studies showing an augmented microglia/macrophage reaction against grafted iPSCs in a motoneuron injury model^26^.

Our earlier study has shown that neuroectodermal cells grafted into the injured spinal cord were able to induce functional recovery due to a secretion mechanism by the transplanted cells^25,27,28^. The effect was exerted by the undifferentiated stem cells soon after grafting in a narrow time window of a few days. In the present study we found a similar phenomenon, ie. intraspinally grafted hiPSCs produced the neurotrophic factor GDNF, the anti-inflammatory cytokine IL-10 and the proinflammatory chemokine MIP1-alpha 1 week after grafting. The expression of these factors by grafted hiPSCs indicates a strong signaling and modulatory process in the injured spinal cord.

GDNF and IL-10 have a potent survival effect on injured neurons after spinal cord injury and reduce secondary damage inflammation and improve motor function^56–61^. Previous reports have shown, that cellular GDNF delivery promotes sensory and proriospinal axonal elongation following spinal cord injury and GDNF overexpression by graft hiPSC-derived NPCs increased the differentiation toward a neuronal fate^62–65^. Here we provided evidence for considerable axon sprouting in the close vicinity of the graft or among the transplanted cells 1 week after grafting. Moreover, IL-10 treatment reportedly induces functional improvement following SCI, promotes neuronal survival and increases axon sparing^57,66^. The exact role of MIP1-alpha is not fully understood in the injured CNS, however, results from our laboratory earlier suggested that it may have a modulatory effect after SCI^26,27^.

Earlier our laboratory and others have shown that stem cells have a functional multipotency feature that allows them to adapt to the lesion environment^25–28,67,68^. Our findings support our previous observations that the grafted cells are able to change the factor production after a short period of time following grafting and this suggests the presence of a strong communicative interaction between the injured host tissue and the grafted cells, leading to the release of a “lesion-induced secretome” by the grafted cells.

## Materials and method

### Statement of ethical approval

The experiments were carried out with the approval of the Committee for Animal Experiments at the University of Szeged regarding the care and use of animals for experimental procedures. All the procedures were carried out in full accordance with the Helsinki Declaration on Animal Rights. Adequate care was taken to minimize pain and discomfort. Animals were given food and water ad libitum.

### Maintenance of SB5 hiPSC line

In this study the SB5 hiPSC line was used^22^, at passage 13 (p13) in the transplantation experiments. Cells were grown on Matrigel (BD Biosciences) coated 6-well plates (Nunc) in mTESR-1 medium (Stem Cell Technologies), following the manufacturer’s instructions. Cells were cultured at 37 °C in humidified atmosphere containing 5% CO_2_ and passaged once every week using Dispase (Stem Cell Technologies) treatment.

### Immunocytochemistry of pluripotency and lineage markers

The expression of pluripotency and germ layer markers was analysed using conventional immunocytochemical staining protocol. The cells were fixed in 4% PFA (20 min, RT), permeabilized with 0.1% Triton X-100 (5 min) and blocked in 1% bovine serum albumin (BSA) containing PBS (1 h, RT). The cells were incubated with primary antibodies overnight at 4 °C: goat anti-NANOG (1:100, AF1997, R&D Systems), mouse anti-OCT3/4 (1:50, sc-5279, Santa Cruz Biotechnology), goat anti-SOX2 (1:100, sc-17319, Santa Cruz Biotechnology), mouse anti-SSEA4 (1:50, sc-59368, Santa Cruz Biotechnology), rabbit anti-PDX1 (1:500, ab47267, Abcam), mouse anti-GATA4 (1:50, sc-25310, Santa Cruz Biotechnology), rabbit anti-Brachyury (1:50, sc-20109, Santa Cruz Biotechnology), mouse anti-Tropomyosin (TPM2) (1:400, T2780, Sigma-Aldrich), rabbit anti-Musashi (MSI1) (1:200, AB5977, Merck-Millipore), mouse anti-NESTIN (1:500, MAB5326, Merck-Millipore), rabbit anti-TUBB3 (1:500, PRB-435P, Covance), mouse anti-MAP2 (1:500, MAB3418, Merck-Millipore). The immune reaction was completed by Alexa Fluor 568 donkey anti-mouse IgG (1:2000, A10037, Thermo Fisher Scientific), Alexa Fluor 568 donkey anti-rabbit (1:2000, A10042, Thermo Fisher Scientific) and Cy3-conjugated donkey anti-goat (1:100, 705–165-147, Jackson Immuno Research). For nuclei counterstaining 0.2 µg/ml DAPI (20 min, RT) was used. The cells were observed under a fluorescence microscope equipped with a 3D imaging module (AxioImager system with ApoTome, Carl Zeiss MicroImaging) controlled by AxioVision 4.8.1 Microscope software (Carl Zeiss MicroImaging).

### Cell viability assessment

Cell viability was determined by using Trypan Blue solution (T8154, Sigma-Aldrich), and Countess II FL Automated Cell Counter (Thermo Fisher Scientific). Harvested cells were resuspended in 1 ml mTESR-1 medium, then 10 µL cell suspension was removed and mixed with 10 µL of Trypan Blue and the mixture was pipetted into a disposable Countess chamber slide and counts were determined. Cell viability was measured 5 and 30 min after cell harvest.

### Spinal cord injury model

All together 80 female Fischer 344 rats (Biological Services, University of Szeged, 180–220 g body weight) were used. This rat strain has poor susceptibility for developing inflammatory reactions^69^, so ideal for xenotransplantation experiments.

All of the operations were carried out under deep ketamine-xylazine anaesthesia (ketamine hydrochloride [Ketavet, 110 mg/kg body weight]; xylazine [Rompun, 12 mg/kg body weight]) and sterile precautions. The surgical area was shaved and disinfected with 70% ethanol and povidone-iodine (Betadine). A midline incision was made at the caudal thoracic area (T6–T12), and the skin and superficial back muscles were retracted. Laminectomy was performed at the T11 vertebral level, the dura mater was exposed and the spinal cord was contused using an Infinity Horizon impactor (IH-0400, PSI LLC), applying 150 kdyn force (moderate injury, Supplementary Fig. S10). The wounds were then sutured in layers and the animals were given postoperative analgesia and saline (0.9%; 5 ml) to prevent dehydration and received meloxicam (Metacam; 0.5 mg/kg body weight, Boehringer Ingelheim Vetmedica). Animals were allowed to recover and housed in standard rat cages at a controlled room temperature. Their bladders were manually expressed three times daily until return of reflexive bladder control. All animals were allowed to survive 2, 3, 5 or 9 weeks after injury.

### Transplantation of hiPSCs and the experimental groups

At 7 days after injury, all injured rats were block randomized into four experimental groups based on their Basso, Beattie, and Bresnahan open field locomotor score (BBB analysis^70^) to ensure equivalent deficits across the experimental groups before starting the treatment. One week after injury, SB5 hiPSCs were transplanted intravenously or intraspinally (depending on the experimental setup). One-week delay of the transplantation was applied based on the results of Péron et al. This study has shown that such delay of the transplantation significantly enhances the survival and proliferation of the grafted cells^71^. In the case of intravenous administration 1 × 10^6^ cells (delivered in 250 μl mTESR-1 medium) were injected in the tail vein, while intraspinally 5 × 10^5^ cells (in 2 µl mTESR-1) were slowly deliver into the lesion cavity, through the use of Hamilton pipette. Control animals received mTESR-1 medium only intravenously (250 µl) or intraspinally (2 µl) one week after injury. The following experimental groups were set up in this study (Supplementary Fig. S11):

- *group 1) medium-iv* medium injected intravenously 1 week after injury (control) n: 12
- *group 2) medium-isp* medium injected intraspinally 1 week after injury (control) n: 16
- *group 3) SB5-iv* hiPSCs transplanted intravenously 1 week after injury n: 24
- *group 4) SB5-isp* hiPSCs transplanted intraspinally 1 week after injury n: 28

### Retrograde labeling

Eight weeks after injury four animals in each group were deeply anaesthetized as described above. laminectomies were made at the T13–L1 vertebral level (corresponding to the L2–L4 spinal level) to expose the upper lumbosacral enlargement. The L3 spinal segment was identified and a right hemisection was performed. Fast Blue (FB) crystals (Dr. Illing Plastics GmbH) were placed into the gap, the dura flap was placed to the hemisection area and the wound was closed in. Rats were kept alive for seven days after the labeling, then they were re-anaesthetized and perfused transcardially. Cryostat sections taken from the brain, brainstem and spinal cord were mounted onto gelatinized slides. The number of FB-positive cells was determined using an epifluorescent microscope (BX-41, Olympus).

### Sacrifice of animals and tissue preparation

At the end of each experimental paradigm (2, 3, 5 and 9 weeks after injury), rats were euthanized by overdose of ketamine-xylazine and perfused transcardially with saline containing heparin followed by 4% paraformaldehyde (PFA) in 0.1 mol/l phosphate buffer (pH 7.4) (all from VWR International). The spinal cord, brainstem and the brain of the animals were carefully dissected and placed into 4% buffered PFA for one day. The fixed tissues were cryoprotected in 30% sucrose in PBS containing 0.01% sodium-azide at 4 °C until being embedded in Shandon Cryomatrix gel (Thermo Fisher Scientific). Parallel or serial transverse (25 μm or 30 μm thick) and longitudinal (16 μm thick) sections were cut on a cryostat (CM 1850, Leica) and mounted onto gelatine-coated glass slides. These methods were according to our previous publications^25–27^.

### Quantitative assessment of the retrogradely labeled neurons

The number of retrogradely labeled neurons was determined according to the method published by Bunge et al*.*^72^ Serial transverse Sections (30 µm thick) were taken from the T5, T1, C6 and C2 spinal segments and every 5th or every 10th coronal Sections (30 µm thick) was used from the brainstem or from the cerebral cortex, respectively. In the case of the spinal cord serial sections, the FB-labeled neurons were mapped and their location was compared to that of the labeled neurons in the neighbouring sections. Thus, double counting of the same neuron was avoided.

### Quantification of cystic area and tissue sparing

Every second transverse section from the T7-L1 segments containing the lesion cavity was stained with cresyl-violet (1% aqueous cresyl-violet solution, C-1791, Sigma-Aldrich) (n = 4 in each group).

The border between the intact tissue and the lesion cavity composed of small cysts was defined. The whole cystic cross-sectional area (lesion cavity area) at the level of the epicentre was determined as follows: the number of pixels of the reference area (1 mm^2^) and that of the cystic area was computed through the use of the NIH ImageJ analysis software (imagej.nih.gov/ij). The pixel number of the cystic area was divided by that of the reference area and the result was expressed in mm^2^. The percentage of spared tissue was determined in a similar manner. Briefly, the number of pixels of the spared tissue was measured at the epicentre (0) and 0.5, 1.0, 1.5, and 2.0 mm rostrally and caudally from it. Identical spinal cord segments of intact animals were used as reference values. The amount of spared tissue in the lesioned animals was given as percentage of intact spinal cord values.

### Immunocytochemistry, immuno- and lectin histochemistry

Nonspecific binding sites were subsequently blocked with 3% normal donkey, goat or horse serum. Primary antibodies and lectin were used as follows: mouse anti-SSEA4 stage-specific embryonic antigen-4, 1:200, MAB1435, R&D Systems), mouse anti-SC-101 (human nuclear marker, 1:500, Y40400, Clontech Laboratories), mouse anti-SC-121 (human cytoplasmatic marker, 1:500, Y40410, Clontech Laboratories), rabbit anti-Ki-67 (1:500, ab1667, Abcam), rabbit anti-TUBB3 (1:500, ab18207, Abcam), mouse anti-TUBB3 (1:500, ab7751, Abcam), rabbit anti-NF200kD (1:500, ab8135, Abcam), rabbit anti-GFAP (1:400, 18-0063, Thermo Fisher Scientific), rat anti-MOG (1:200, MAB2439, R&D Systems), goat anti-5-HT (1:500, ab66047, Abcam), mouse anti-CS-56 (1:200, C8035, Sigma-Aldrich), biotinylated Griffonia Simplicifolia isolectin B4 (GSA-B4, 1:200, B1205, Vector Laboratories), rabbit anti-BDNF (1:200, ab72439, Abcam), rabbit anti-GDNF (1:200, ab18956, Abcam), rabbit anti-IL-1-alpha (1:150, 250,715, Abbiotech), mouse anti-IL-6 (1:250, ab9324, Abcam), rabbit anti-IL-10 (1:150, E92171, Enogene), rabbit anti-MIP-1-alpha (1:200, ab9781, Abcam), rabbit anti-TNF-alpha (1:150, ab6671, Abcam), rabbit anti-NT-4/5 (1:200, 250,792, Abbiotech), rabbit anti-VEGF (1:100, sc-507, Santa Cruz Biotechnology) and rabbit anti-PDGF-A (1:100, sc-7958, Santa Cruz Biotechnology). The following secondary antibodies were used: biotinylated goat anti-rat IgG (1:200, BA-9400, Vector Laboratories). The immune reaction was completed by Alexa Fluor 594 donkey anti-mouse (1:600, A21203, Thermo Fisher Scientific), Alexa Fluor 488 goat anti-rabbit (1:600, A11008, Thermo Fisher Scientific), Alexa Fluor 546 donkey anti-rabbit (1:600, A11040, Thermo Fisher Scientific), Alexa Fluor 488 donkey anti-goat (1:600, A11055, Thermo Fisher Scientific), Streptavidin Alexa Fluor 488 Conjugate (1:600, S-11223, Thermo Fischer Scientific), Streptavidin Alexa Fluor 405 Conjugate (1:600, S-32351, Thermo Fischer Scientific,). The sections were covered using Vectashield mounting medium containing DAPI (1.5 µg/ml; H-1000-10, Vector Laboratories), which labeled the nuclei of the cells. Negative controls for the secondary antibodies were performed by omitting the primary antibodies. Immunoreactive sections were viewed by a BX-41 epifluorescent microscope equipped with a DP-74 digital camera or Olympus FV-10i-W compact confocal microscope system (Olympus).

### Analysis of the hiPSC differentiation engrafted in the spinal cord

These analyses were performed based on previously described methods^46,73^. To quantify the differentiation pattern of engrafted cells (n = 4 in each transplanted group), we immunostained sagittal sections of the spinal cords containing hiPSCs and derivatives at 1 and 2 weeks after transplantation. For Ki-67 labeling, we randomly selected three tissue sections that were 90 µm apart from each other. Images taken by confocal microscopy at 60× magnification were used for quantitative analysis. The numbers of SC-101 or SC-121/DAPI-positive cells were counted in 5 randomly selected fields per section. Next, those SC-101 or SC-121/DAPI-positive cells were counted that were colabeled with Ki-67 or TUBB3 and their percentages were given. SSEA-4 quantifiaction, the number of DAPI-positive cells were counted. Next the DAPI/SSEA-4-colabeled cells were counted and their ratio was given.

### Analysis of biodistribution of intravenously applied hiPSCs

The spinal cord, lungs, liver and spleen were collected 7 days after cell injection (n = 4). The tissues were postfixed for 1 day with 4% PFA, cryoprotected and embedded in Shandon Cryomatrix gel. The 16 or 30 µm thick tissue sections were stained with anti-SC-121 and DAPI. Images were taken with an Olympus BX-41 epifluorescence microscope equipped with a DP-74 digital camera using the Cell Sense software (Olympus).

### Quantification of CS-56, GFAP, GSA-B4 expression after the injury

To assess the density of GFAP + , GSA-B4 + and CS-56 + reactivities in spinal cords of injured and treated animals (n = 4 in each group), two sagittal Sects. (150 µm apart from each other) containing the lesion cavity were analysed for each marker, 9 weeks after injury. Microphotographs were taken using an Olympus BX-41 epifluorescence microscope equipped with a DP-74 digital camera and the whole spinal cord section area including the cavity and a 2 mm long extension of the tissue rostrally and caudally from the cavity ends was analysed using ImageJ software. The background intensity of unstained samples was individually subtracted from the intensity of treated sections. To correct for interanimal variations in the immunolabeling efficiency, we normalized the intensity of the immunolabeled tissue to the same section (uninjured area) 4 mm rostral to tip of the lesion area/grafted area. Data were expressed as fold increase immunointensity normalized to uninjured value.

### Quantification of NF-200 and TUBB3 expression after the injury

In the case of NF-200kD and TUBB3 we used the method as mentioned above (2 and 9 weeks after injury), but the extension was only 200 µm from the graft-host (in *SB5-isp* group) or the spared-lesion (in *medium-isp* group) border. The intensity of immunolabeled area was normalized to the plain injured spinal cord. Data were expressed as fold increase immunointensity normalized to plain injured value.

### Quantification of 5-HT expression caudal to the injury

Two sagittal sections of the spinal cord (*medium-isp*, SB5-isp and plain injured spinal cord) were photographed at 20× primary magnification using an Olympus BX-51 epifluorescence microscope equipped with a DP-74 digital camera at four distances caudally from the cavity ends (500, 1000, 1500 and 2000 µm; in an area of 100 µm × 500 µm in each distance). Using ImageJ Software (NIH), relative density of 5-HT immunoreactivity was measured in entire sagittal section of the spinal cord. Background intensity unstained samples was subtracted from the intensity value to correct for nonspecific reactions. The density of the immune-labeled tissue was normalized to the plain injured spinal cord at all examined distances caudally to the lesion. Data were expressed as fold increase immunointensity normalized to plain injured value.

### Quantification of factors produced by the grafted cells

Quantification of the factor expression by grafted cells was performed using ImageJ software according to Tieng et al.^74,75^ Briefly, images were taken by confocal microscopy at 120× magnification. The numbers of IL-10-, GDNF- and MIP1-alpha-immunoreactive pixels were measured in 5 randomly selected area/animal. Data was normalized with DAPI positive nuclei number. Data were expressed as percentage of marker/DAPI ratio.

### BBB open field locomotor score

The Basso, Beattie, Bresnahan (BBB) locomotor rating scale^70^ was performed 3 days and every 1 week after injury up to 8 weeks (n = 8 in each group). Two observers, unaware of experimental procedures tested the animals. Rats were assessed in an open field (150 × 100 cm) for 4 min at a similar time of day for each testing. We randomly allocated the injured animals into four experimental groups in the manner that all groups consisted of animals with comparable range of BBB scores as well as group average. This randomization ensured the presence of equivalent locomotor deficits across the groups before the beginning of treatment.

### Analysis of locomotion pattern

Between the 4th and the 8th postoperative week video-based kinematic analysis was carried out (n = 8 in each group). The hair of the rats was shaved off from the hind limbs and the skin was marked by a black pen above the major joints. We used a plexiglass runway equipped with a mirror system to be able to record the position of the hind limb from both lateral and rear-view aspects. Two high resolution and high-speed cameras (GoPro Hero 3 + Black Edition, GoPro; DFK 22AUC03, The Imaging Source, www.gopro.com) were used to during 3 to 4 step cycles. The animals were trained prior to the measurements to walk from one end of the runway to the other reaching a shelter and were tested every week postoperatively. By comparing specific single video frames we measured six different parameters to get detailed information on the recovery. The lateral placing parameter is the angle enclosed by the axis of the tarsus and the longitudinal axis of the animal. We measured this parameter from ventral mirror view. The Toe off angle (TOA) is the angle enclosed by the floor plate and the line formed by the tarsal and metatarsal bones. Ankle flexion (AF) which is an angle enclosed by the tarsus and the tibia was measured. Knee flexion (KF) has been determined as the angle enclosed by the tibia and the femur at the first moment of the stance phase. We measured these last 3 parameters from lateral view. The angle enclosed by the metatarsus and the surface and the tarsus-surface angle were observed from rear-view aspect. A methodological manuscript detailing the description of the above set up^76^ and analysis is currently being prepared for publication.

### Image processing and statistical analysis

Graphs were created by Microsoft Office Pro Plus 2016 (Microsoft, www.office.com). Graphs and representative images were further processed using the GNU Image Manipulation Program (GIMP 2.10.0, www.gimp.org).

All results were analyzed using SPSS version 24.0 (IBM). BBB scores and analysis of locomotion pattern for each group were analysed using repeated measures two-way ANOVA. The Tukey's all pairwise multiple comparison procedure was used to correct for multiple comparisons. Comparisons of quantitative immunohistochemistry data were carried out by using the Student’s t-test or one-way ANOVA with Tukey’s post-hoc test. Data are presented as mean + Standard Error of Mean (SEM), and *p* < 0.05 was considered to be significant.

## Supplementary Information

Supplementary Information.
