# Supplementary material for: Grafted human induced pluripotent stem cells improve the outcome of spinal cord injury: modulation of the lesion microenvironment
Source: Sci Rep. 2020 Dec 29;10:22414. doi: 10.1038/s41598-020-79846-2 (PMC7772333; doi:10.1038/s41598-020-79846-2)
Supplement: Supplementary file 1 — Supplementary Information. [file 41598_2020_79846_MOESM1_ESM.docx]

Grafted human induced pluripotent stem cells improve the outcome of spinal cord injury: modulation of the lesion microenvironment

Tamás Bellák^1,2^, Zoltán Fekécs^1^, Dénes Török^1^, Zsuzsanna Táncos^2^, Csilla Nemes^2,†^, Zsófia Tézsla^1^, László Gál^1^, Suchitra Polgári^2^, Julianna Kobolák^2^, András Dinnyés^2,3,4^, Antal Nógrádi^1*^, Krisztián Pajer^1^

^1^Department of Anatomy, Histology and Embryology, Faculty of Medicine, University of Szeged, Szeged, Hungary

^2^BioTalentum Ltd., Gödöllő, Hungary

^3^HCEMM-USZ StemCell Research Group, Szeged, Hungary

^4^Research Institute of Translational Biomedicine, Department of Dermatology and Allergology, University of Szeged, Hungary

^†^ current address: Military Hospital, State Health Centre, Department of Diagnostic Laboratory, Budapest, Hungary

*corresponding author:

Antal Nógrádi

Department of Anatomy, Histology and Embryology

Faculty of Medicine

University of Szeged

H-6724, Szeged, Hungary

Kossuth Lajos sgt. 40.

Tel.: +36-62-342855

Fax: +36-62-342856

E-mail: [nogradi.antal@med.u-szeged.hu](mailto:nogradi.antal@med.u-szeged.hu)

**Supplementary Information**

**
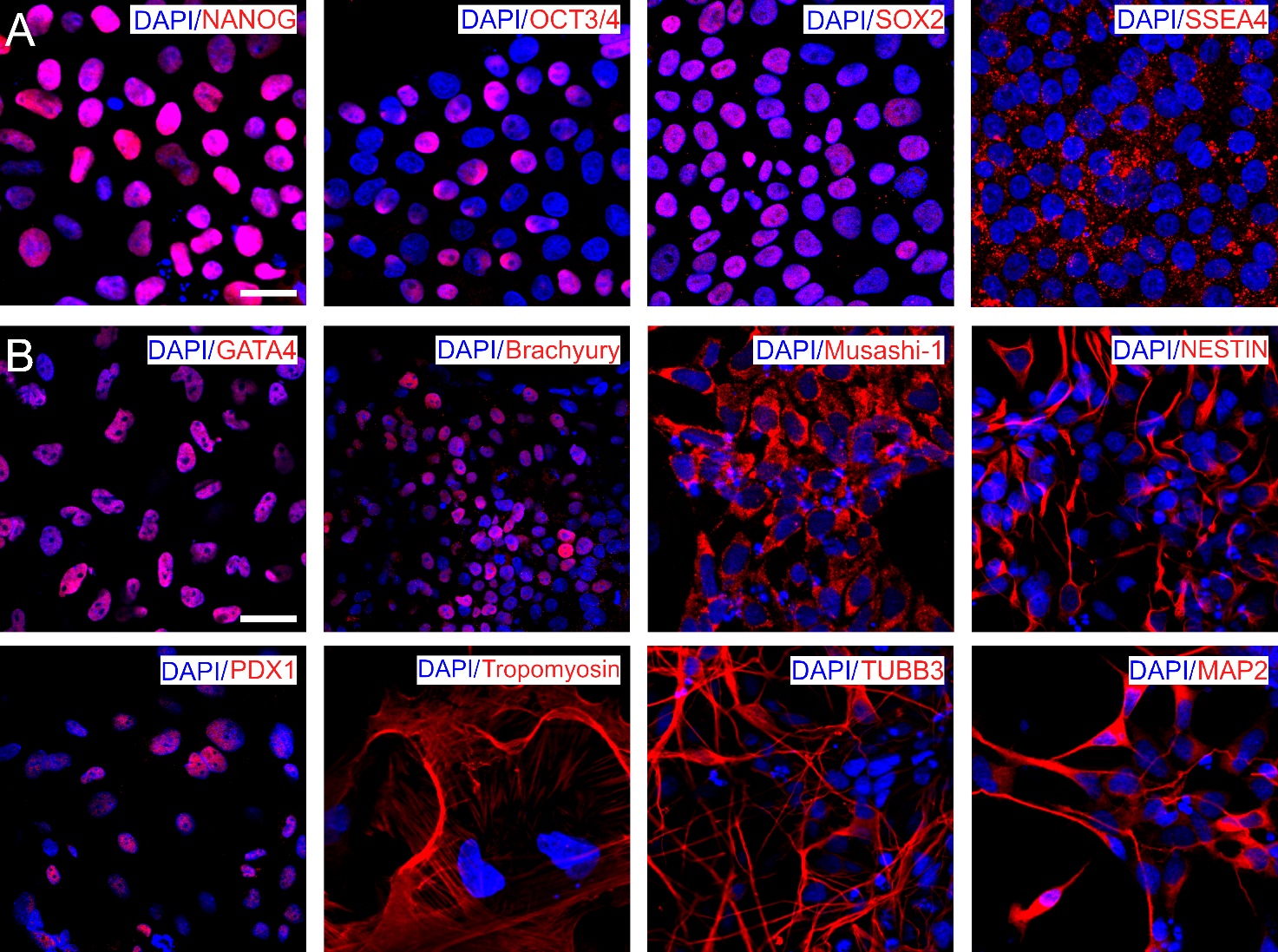
**

**Supplementary Figure S1 In vitro characterization of the SB5 hiPSC line.** (A) hiPSCs expressed the pluripotency markers NANOG, OCT3/4, SOX2 and SSEA4. (B) Immunofluorescence analysis was used to detect differentiated cells positive for markers representing each embryonic germ layer after spontaneous differentiation of the cells. Cells expressed GATA4, PDX1 (endoderm), Brachyury, Tropomyosin (TPM2) (mesoderm), Musashi-1 (MSI1), NESTIN, TUBB3 and MAP2 (ectoderm) proteins. Nuclei were stained with DAPI (blue). Scale bars: 25 µm. Photos were taken by AxioVision 4.8.1 Microscope system (Carl Zeiss MicroImaging) and were further processed using the GNU Image Manipulation Program (GIMP 2.10.0, www.gimp.org).

**
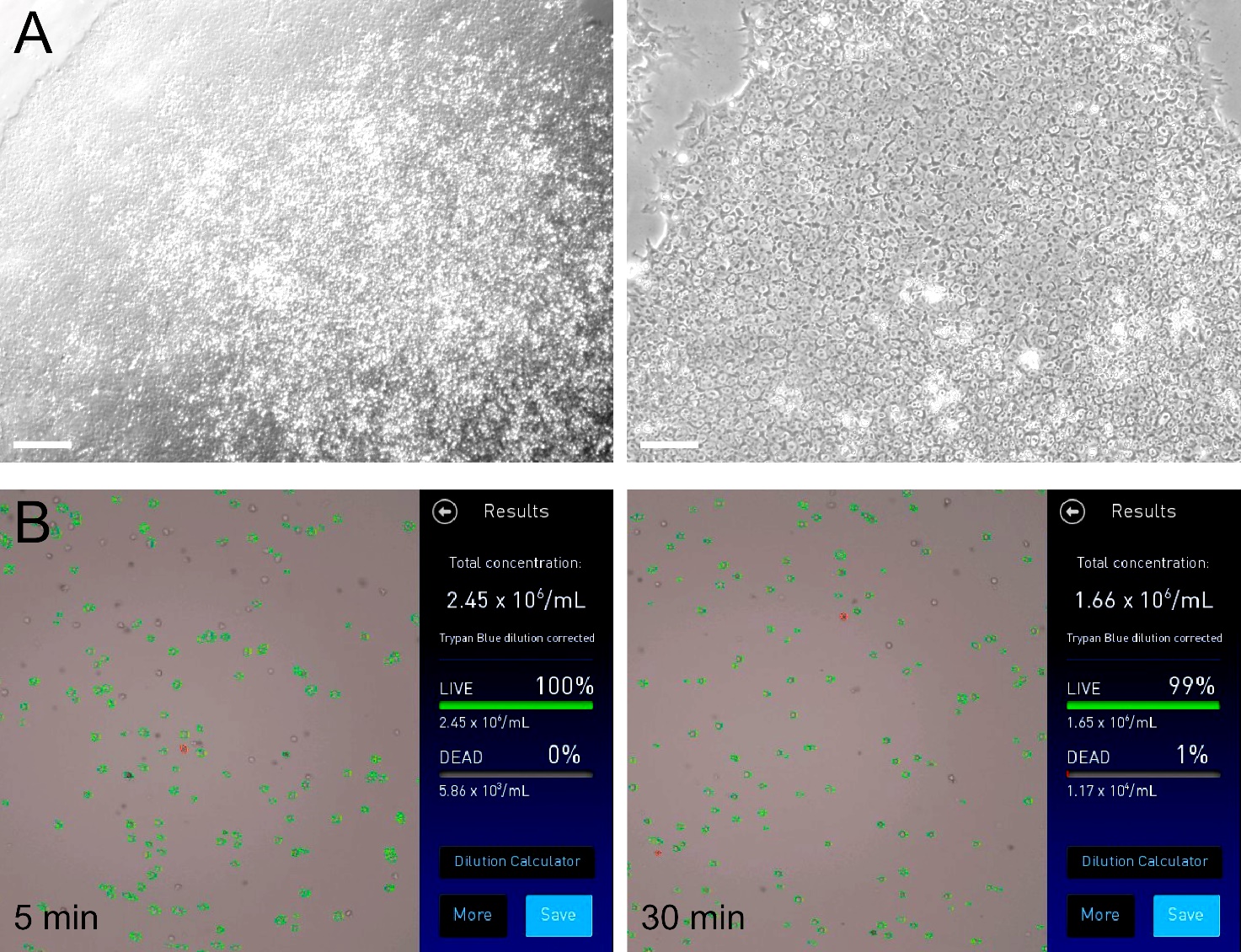
**

**Supplementary Figure S2 Cell viability measurements before transplantation** (A) Representative images of SB5 hiPSCs colonies. (B) The assessment showed 100% (5 min) and 99% (30 min) cell viability after cell harvest. Scale bars: 250 µm and 100 µm.

**
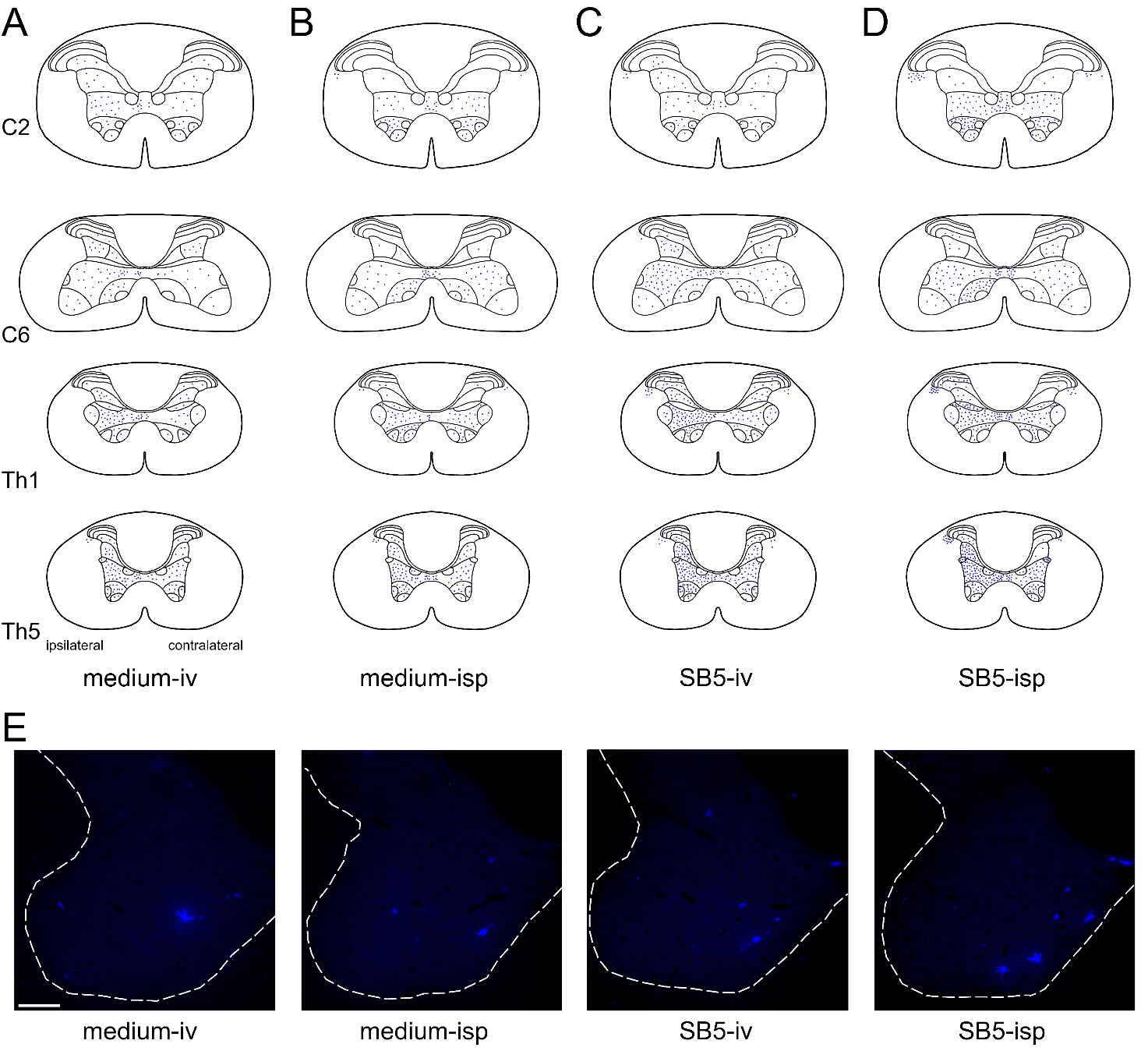
**

**Supplementary Figure S3 The schematic views of various spinal cord segments summarize the localization of retrogradely labelled propriospinal neurons in the expreimental groups.** (A-D) The number of retrogradely labelled neurons was significantly higher in animals that received stem cells (SB5-iv, SB5-isp) compared to their controls (medium-iv, medium-isp). It should be noted that the number of retrogradely traced neurons decreased with the distance from the labeled segment. (E) Representative confocal microscopy images show the FB+ neurons in the C2 spinal segment (ventral horns) in the different experimental groups. Dashed line indicates the border between gray and white matter. Scale bar: 200 µm.


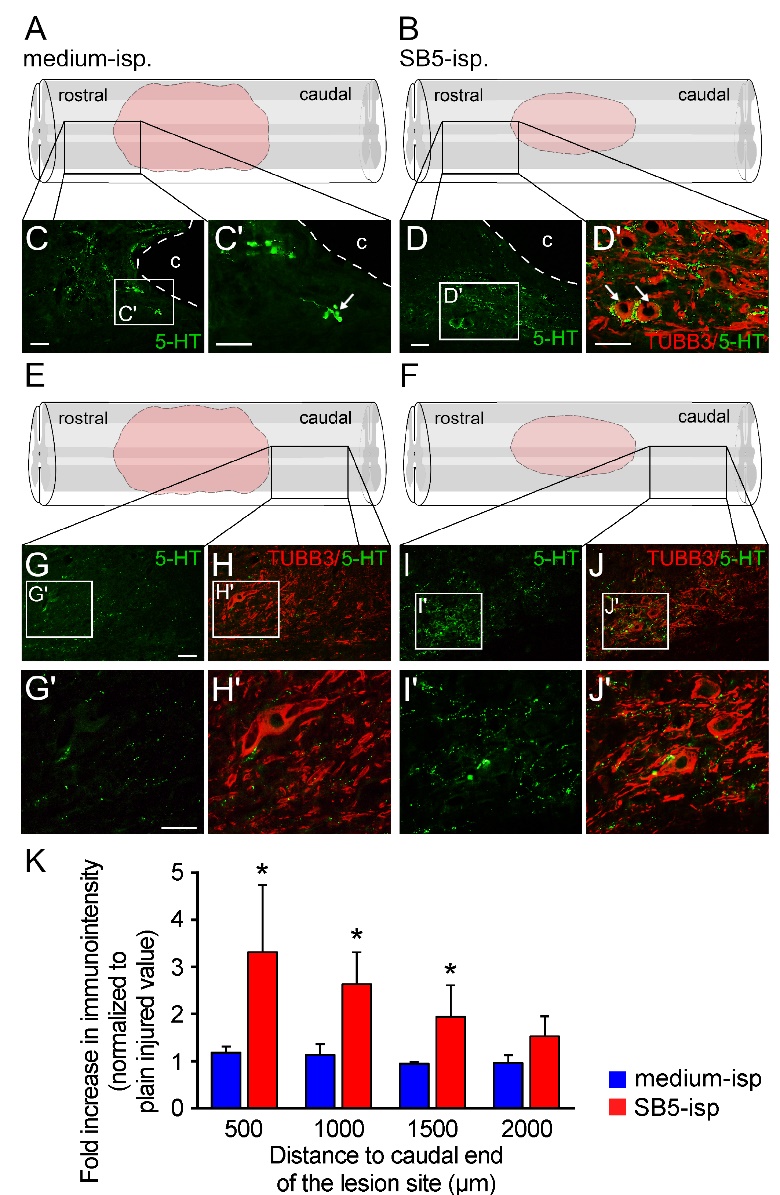


**Supplementary Figure S4** **hiPSC graft promotes the plasticity of serotonergic fibers in contused spinal cord.** (A, C and C’) Longitudinal section of a control spinal cord (*medium-isp*) at a low thoracic region demonstrates that 5-HT-positive fibers reach the cranial end of the cavity. The fibers form aborted endings (see enlarged framed area, C’). (B, D and D’) Well preserved serotonergic fibers can be observed around the neurons at the cranial end of the cavity in grafted rats (*SB5-isp*). (E, G-H’) In contrast to the spinal level at the rostral end of the cavity, 5-HT-immunoreactive fibers are significantly diminished at the caudal end of the cavity in the control group (*medium-isp*). (F, I-J’) Caudally to the injury, a number of 5-HT-positive fibers is depicted in a grafted cord. (K) Quantification of 5-HT immunointensity in control and grafted groups compared to the plain injured spinal cord at all examined distances caudally to the lesion. Arrows show aberrant serotonergic endings in C’. Arrows show intimate interactions between 5-HT-positive fibers and neurons in D’. c= cavity, Scale bars: C, D, G: 25 µm, C’, D’, G’: 25 µm.


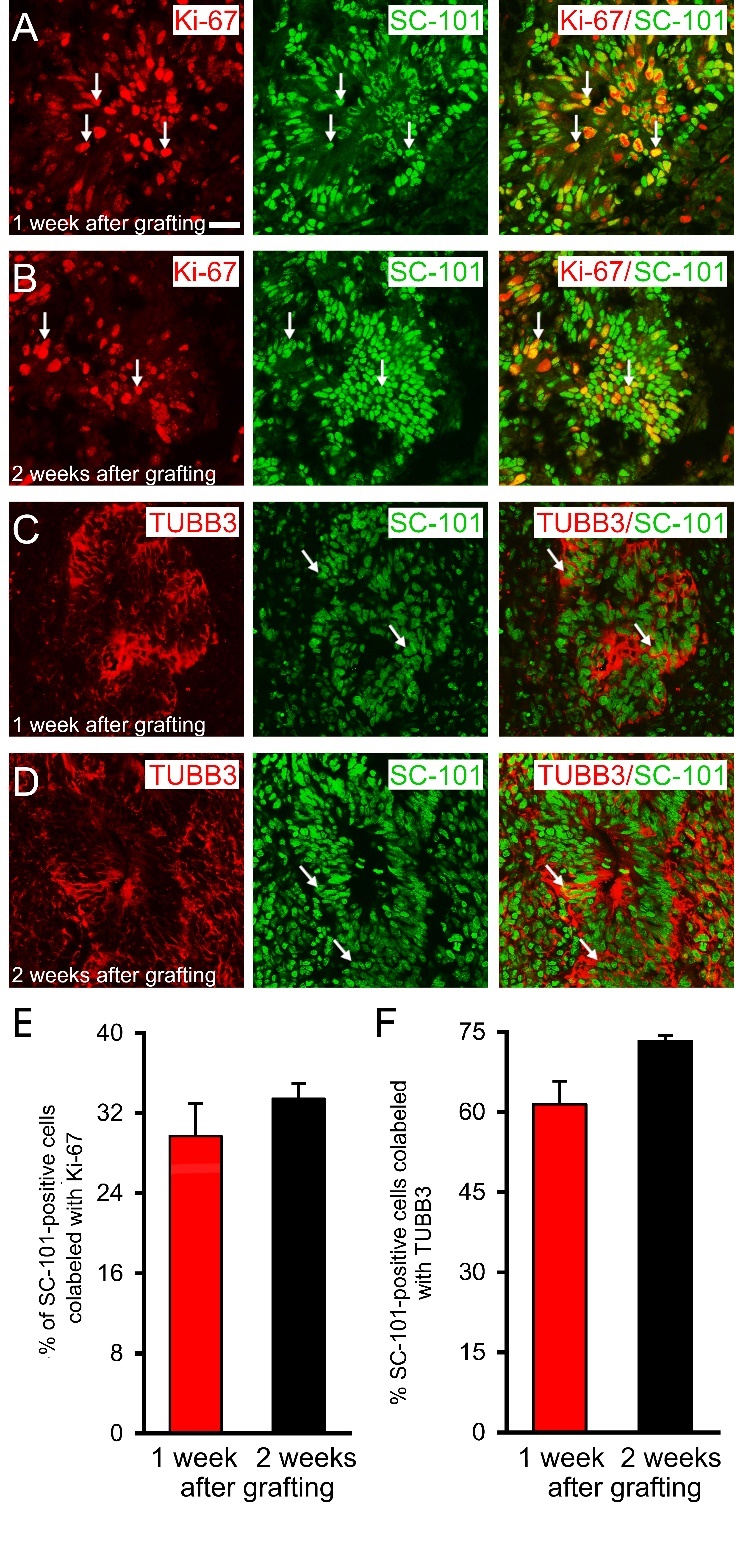


**Supplementary Figure S5 Grafted hiPSCs express Ki-67 and TUBB3 within the injured spinal cord. (A, B)** Confocal images show grafted SC-101-positive hiPSCs (green) colocalized with Ki-67 (red). (C, D) Colabeling of hiPSCs (SC-101, green) with TUBB3 protein (red). (E) Approximately 29.56 ± 3.15 % and 33.36 ± 1.4 % of SC-101-positive cells were colabeled with the Ki-67 1 and 2 weeks after grafting, respectively. (F) Cell quantification showed that the majority of engrafted SC-101-positive cells displayed a neuronal fate identified by TUBB3 expression (62.5 ± 4.26 % and 74.86 ± 1%) 1 and 2 weeks after transplantation, respectively. Arrows show SC-101 positive grafted cells (green) colocalized with Ki-67 (A, B) and TUBB3 (C, D). Scale bar: 20 µm.


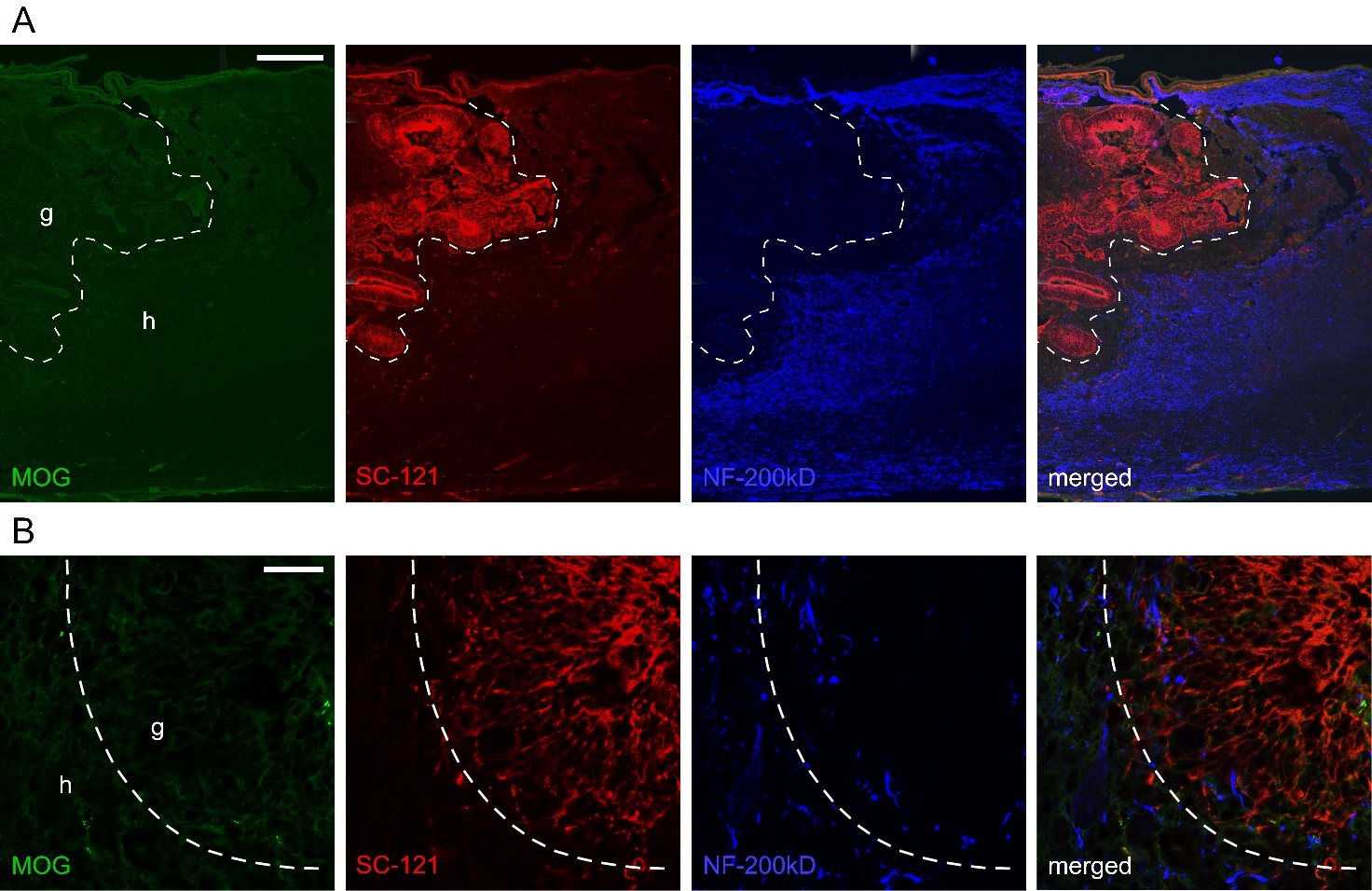


**Supplementary Figure S6 Intraspinally transplanted hiPSC could not differentiate into myelinating oligodendrocytes.** Immunohistological examination at 1 week after transplantation showed the lack of h/mMOG-positive (green) grafted cells, suggesting that the grafted hiPSCs (SC-121, red) were not able to differentiate into mature myelinating oligodendrocytes. Some NF-200kD-positive (blue) host processes were observed among the grafted cells at the host graft interface. Dashed line indicates the graft-host border. Scale bars: A: 100 µm B: 50 µm.


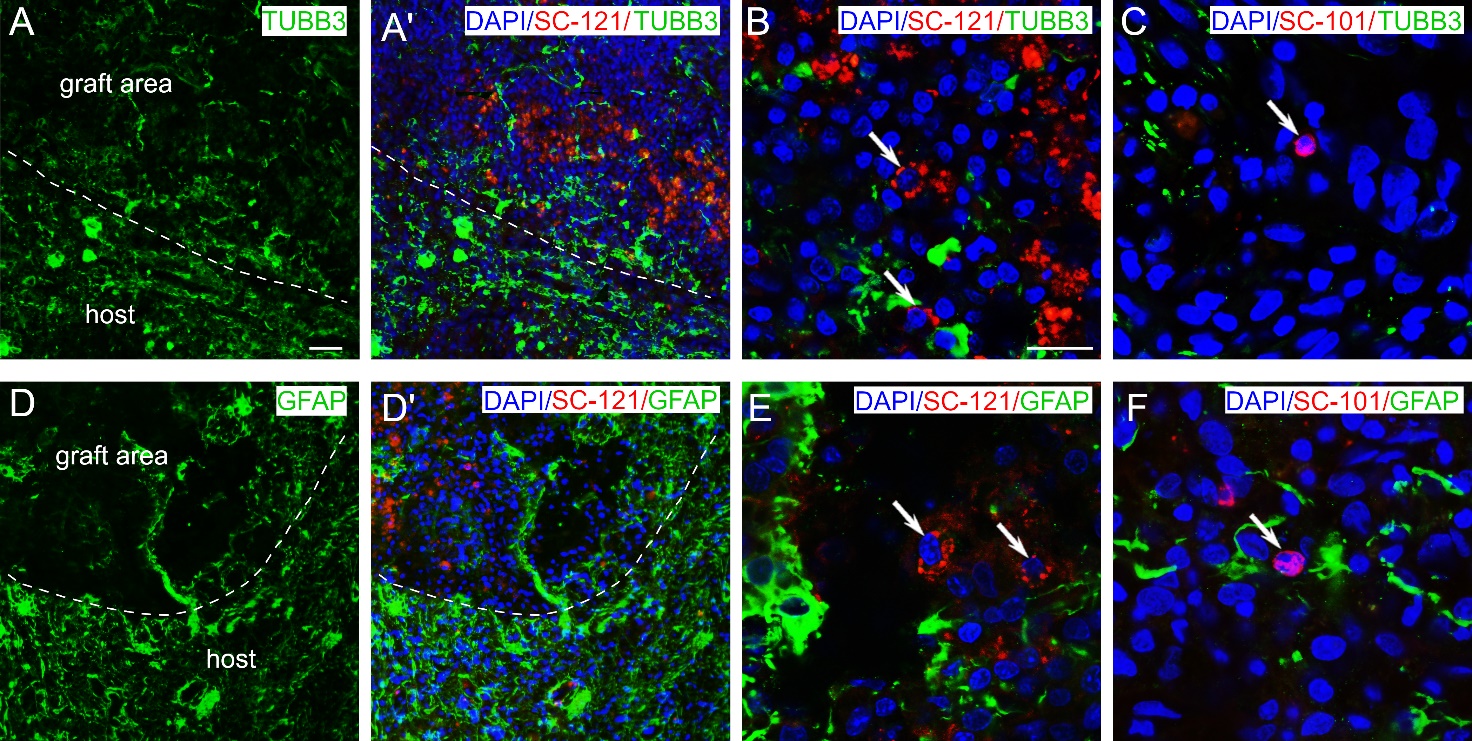


**Supplementary Figure S7 hiPSC transplant disappeared from host injured spinal cord 4 weeks after grafting.** (A-B and D-E) Confocal images of longitudinal sections of grafted spinal cord show SC-121-positive profiles 4 weeks after transplantation. The majority of SC-121 profiles were not positive for TUBB3 or GFAP. (C and F) SC-101-positive immunolabeling was colocalized with DAPI. Arrows show SC-121- (in B and E) and SC-101-positive cells (in C and F). Scale bars: A: 50 µm, B: 25 µm.


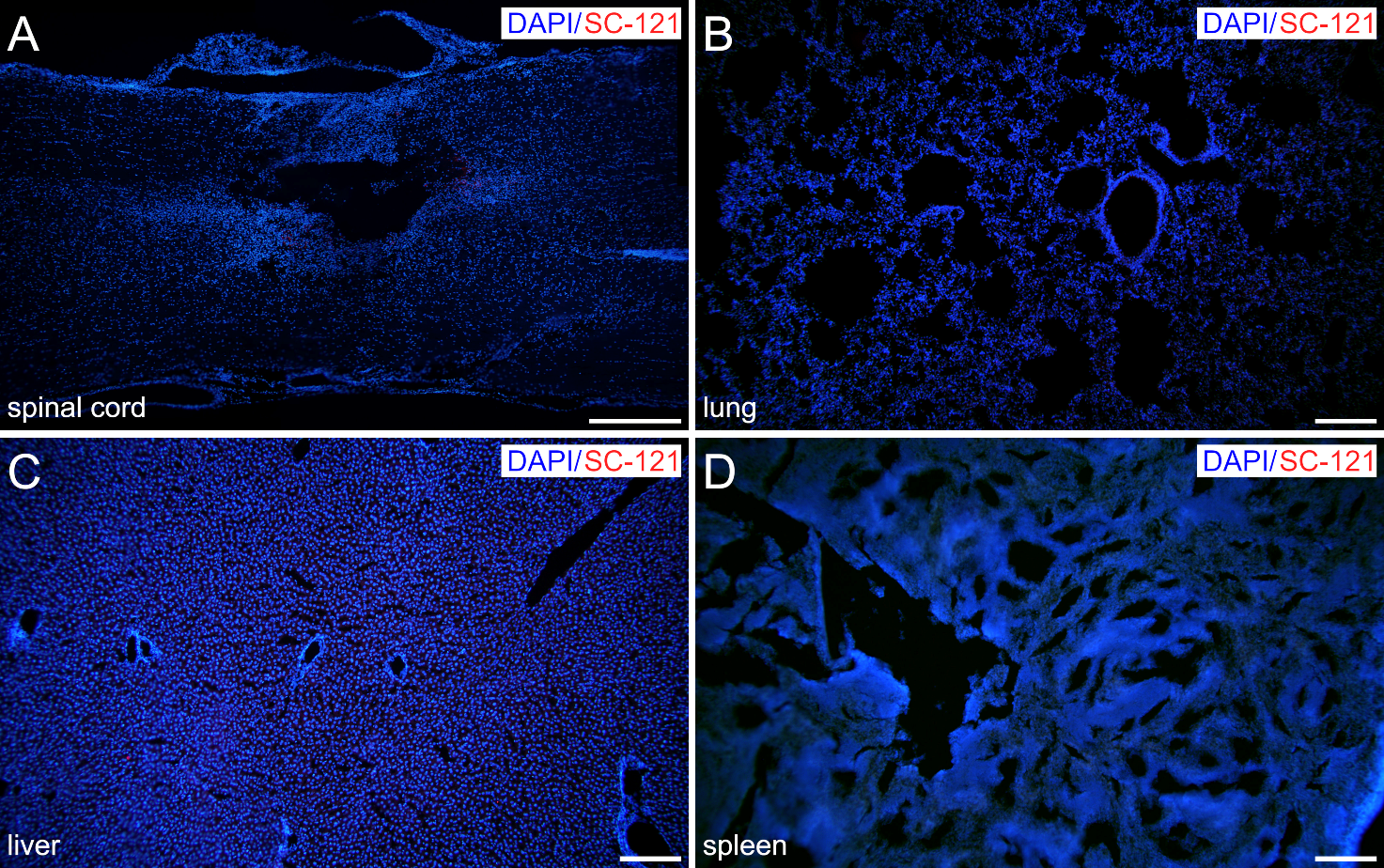


**Supplementary Figure S8 Fate of intravenously transplanted hiPSCs in different organs.** (A-D) Seven days after the intravenous transplantation (*SB5-iv*) the presence of hiPSCs was determined in the spinal cord (A), lung (B), liver (C) and spleen (D). Microphotographs show the SC-121 (human citoplasmatic marker, in red) and DAPI (nuclei marker, blue) stained parasagittal (spinal cord) or cross (lung, liver, spleen) sections. No SC-121-positive cells were detected in the investigated organs that indicates a rapid cell clearance after transplantation. Scale bars: 500 µm (in A) and 100 µm (in B, C, D).

**
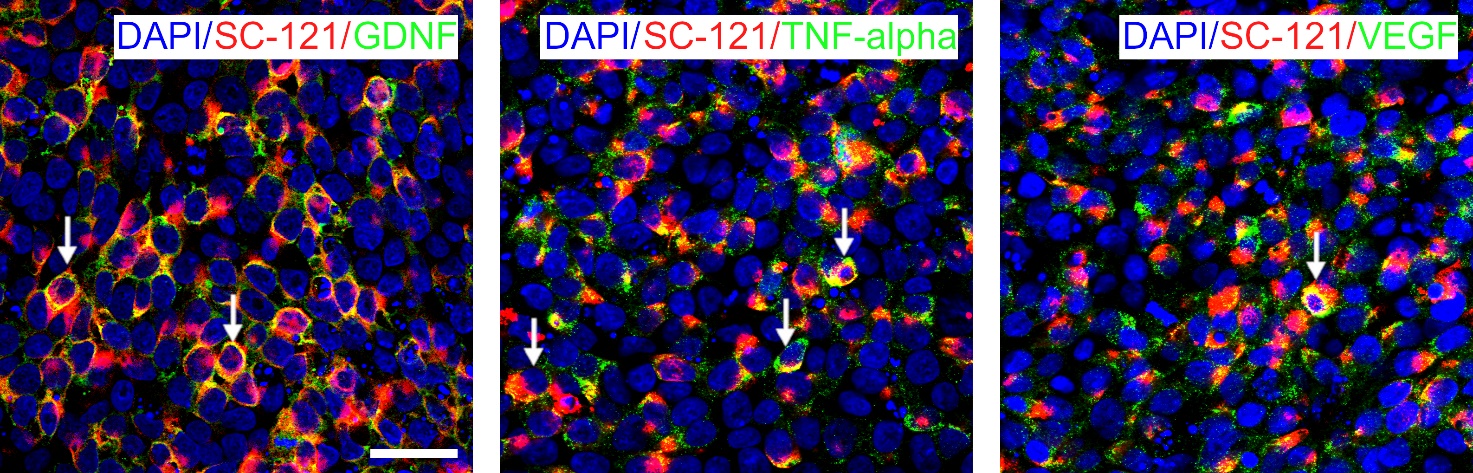
**

**Supplementary Figure S9 Expression of factors produced by hiPSCs in vitro.** GDNF, TNF-alpha and VEGF expressions of hiPSCs were confirmed by immunofluorescence analysis. Arrows show colocalized cells. Scale bar: 50 µm.

**
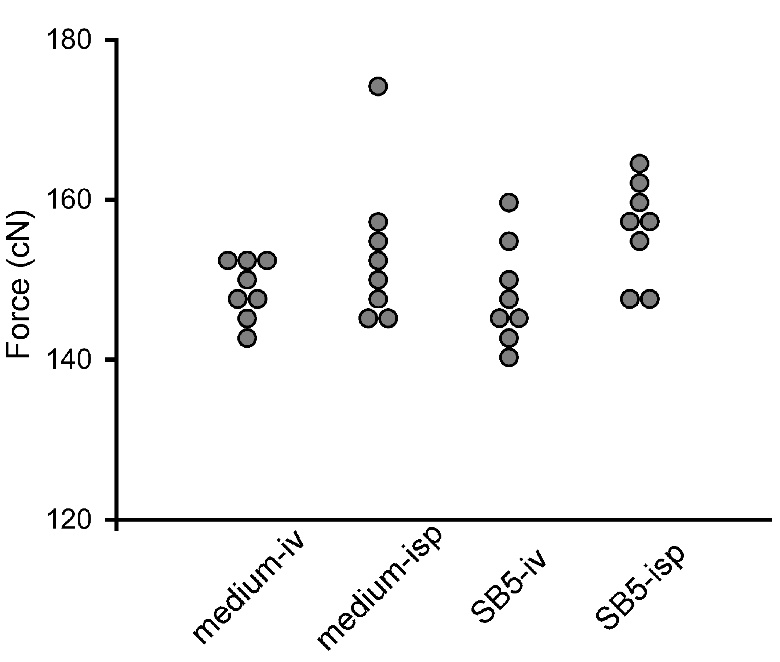
**

**Supplementary Figure S10 Contusion data in the different experimental groups** Each point represents an individual animal with the force applied.

**
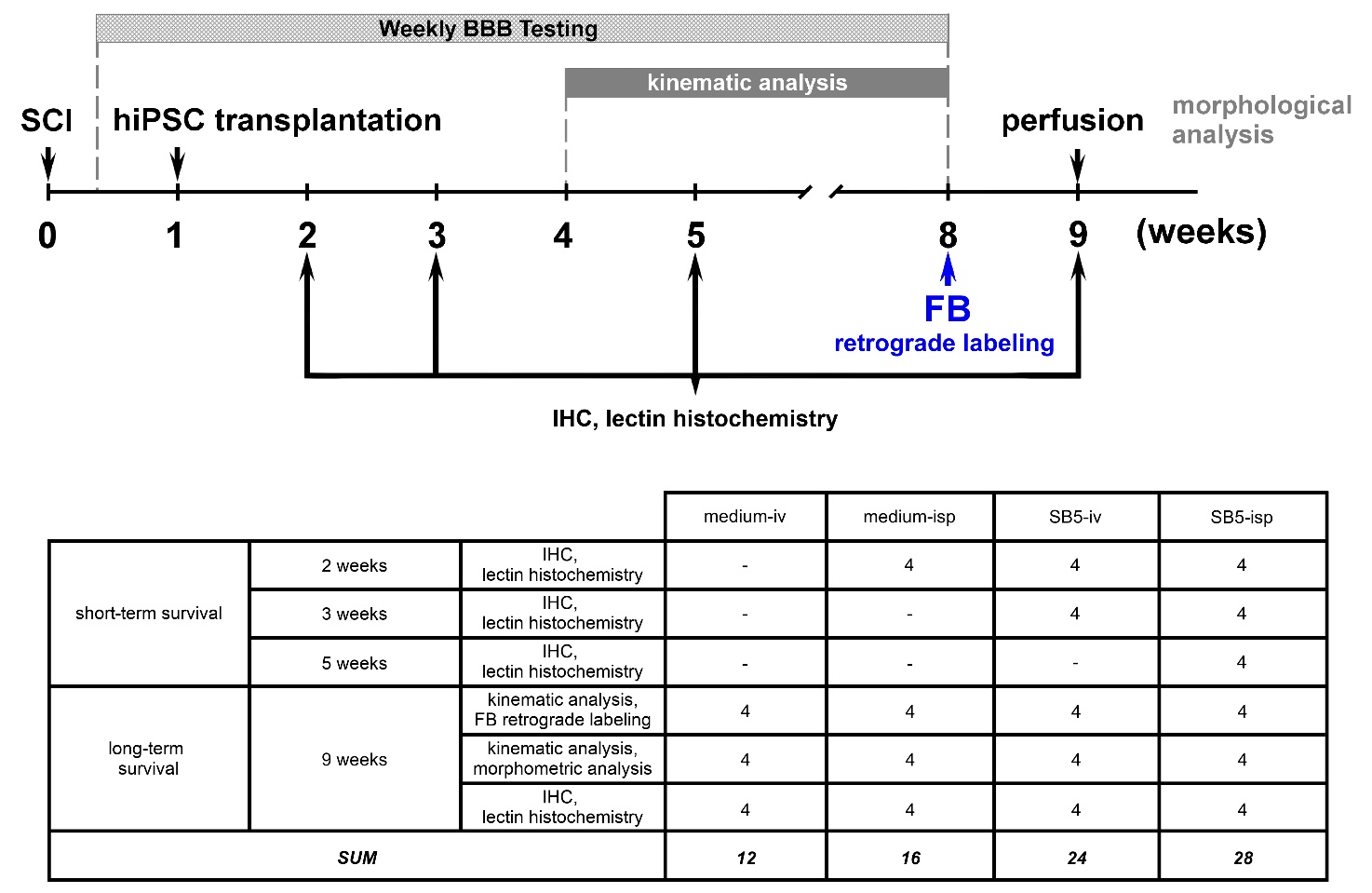
**

**Supplementary Figure S11 Study design illustrating experimental groups, gait and morphological analysis.** hiPSCs were grafted intraspinally or intravenously one week after a thoracic (T11) spinal cord contusion injury. Locomotor analysis of the injured animals was performed by BBB-test and a detailed kinematic analysis system. Two months after the injury the retrograde tracer Fast Blue (FB) was applied distal to the injury to determine the extent of axonal sparing/regeneration. To quantify the differentiation pattern of engrafted cells and investigate the lesion microenvironment (immune)histochemical analyses were performed at week 2, 3, 5 and 9.
